# Electronic softening of degraded LiCoO_2_ enables direct regeneration via ligand-mediated interface modulation

**DOI:** 10.1093/nsr/nwag369

**Published:** 2026-06-13

**Authors:** Jiahuan Zhou, Siyu Zhang, Caichao Wan, Yangyang Liu, Junwei Han, Mohamed H Helal, Ghassab M Al-Mazaideh, Jiang Zhou

**Affiliations:** School of Materials Science and Engineering, Hunan Provincial Key Laboratory of Electronic Packaging and Advanced Functional Materials, Central South University, Changsha 410083, China; School of Materials Science and Engineering, Hunan Provincial Key Laboratory of Electronic Packaging and Advanced Functional Materials, Central South University, Changsha 410083, China; College of Materials and Energy, Central South University of Forestry and Technology, Changsha 410004, China; School of Instrument Science and Technology, Xi’an Jiaotong University, Xi’an 710049, China; School of Minerals Processing and Bioengineering, Central South University, Changsha 410083, China; Center for Scientific Research and Entrepreneurship, Northern Border University, Arar 73213, Saudi Arabia; Department of Pharmaceutical Chemistry, College of Pharmacy, University of Hafr Al-Batin, Hafr Al-Batin 31991, Saudi Arabia; School of Materials Science and Engineering, Hunan Provincial Key Laboratory of Electronic Packaging and Advanced Functional Materials, Central South University, Changsha 410083, China

**Keywords:** direct regeneration, degraded lithium cobalt oxide, electronic structure modulation, acetylacetonate ligand, interface engineering

## Abstract

The direct regeneration of degraded lithium cobalt oxide (LiCoO_2_, LCO) cathodes is pivotal for sustainable battery recycling, yet remains hindered by the intricate lattice defects arising from surface electronic-structure instability. Most existing regeneration strategies rely on compositional compensation while neglecting electronic-level regulation, and lack research on spontaneously formed, electrochemically unfavorable interfaces (rigid interface) that impede ion transport. Herein, we demonstrate a ligand-mediated electronic modulation strategy using a lithium-aluminum acetylacetonate complex (LAAE), where acetylacetonate (acac) functions as an electronic mediator rather than a conventional chelator. Through *π*→*d* orbital coupling with high-valence cobalt sites, acac redistributes Co–O bonding characteristics, creating an electronically compliant surface. This adsorption-induced bond weakening preferentially destabilizes defect-associated Co–O linkages, facilitating the removal of electronically inactive cobalt species and oxygen vacancy passivation, while lowering interfacial ion transport barriers. Simultaneously, lithium replenishment from Liacac-derived species together with Al^3+^ incorporation contributes to stabilizing the layered framework. The regenerated LCO (R-LCO-Al) achieves a near-ideal stoichiometry with suppressed oxygen vacancies and restored structural ordering, delivering a high specific capacity of 179.9 mAh g^−1^ at 0.5 C (3.0–4.5 V) and 87.0% capacity retention after 200 cycles, rivaling commercial LCO. These findings establish electronic-structure modulation as an effective strategy for closed-loop regeneration of degraded oxide cathodes.

## Teaser

A molecular ligand approach restores degraded lithium battery cathodes by tuning their surface chemistry, enabling sustainable direct regeneration of high-performance materials rivaling commercial products.

## INTRODUCTION

The rapidly increasing deployment of lithium-ion batteries (LIBs) in consumer electronics and electric vehicles has led to a growing accumulation of spent batteries, raising pressing concerns regarding resource sustainability and environmental impact [1,2]. Among commercial cathode materials, lithium cobalt oxide (LiCoO_2_, LCO) continues to be widely utilized because of its high operating voltage and superior volumetric energy density. However, when operated under high-voltage conditions above 4.5 V, LCO undergoes pronounced and largely irreversible performance degradation, severely limiting its service lifetime and recyclability [3]. While this degradation is often discussed in terms of structural disorder or compositional imbalance, mounting evidence indicates that such descriptions capture only part of the underlying failure process [4–7].

At a more fundamental level, the degradation of LCO is closely linked to irreversible electronic-structure instability that develops at the surface and near-surface regions during deep delithiation [8]. Repetitive lattice oxygen release and Co^3+^/Co^4+^ redox reactions induce oxygen vacancies and reduced Co species, progressively perturbing the Co–O covalent network [9]. These electronic perturbations induce charge localization and energy-level mismatch between O 2*p* and Co 3*d* states, giving rise to an electronically rigid interface that impedes Li^+^ transport and accelerates further structural decay [10]. Unlike artificially constructed controllable heterogeneous phases, these rigid interfaces form spontaneously during cycling, exhibiting poor structural reliability and higher ion transport barriers. Furthermore, these spontaneously degraded interfacial phases lead to severe heterogeneity in lithium-ion transport, exacerbating issues such as electrode internal stress and abrupt changes in lattice parameters [11]. From this perspective, the irreversibility of LCO degradation arises not simply from the presence of defects, but from the evolution of an unfavorable electronic landscape that is difficult to re-equilibrate once established.

In response to the growing demand for cathode recycling, various direct regeneration strategies have been developed with the aim of restoring degraded LCO while minimizing energy input and secondary pollution [12,13]. Representative approaches include solid-state sintering and chemical lithiation, focused primarily on compositional compensation and structural repair [14,15]. These methods have achieved partial success by addressing thermodynamic or kinetic limitations, such as enhancing Li^+^ diffusion or reducing insertion resistance [16–20]. Nevertheless, most existing strategies implicitly treat degradation as a structural or compositional problem and therefore focus on repairing its observable consequences. The electronic structure of the degraded interface where irreversible failure is initiated and reinforced, remains largely unregulated [21–23]. As a result, regenerated materials often exhibit limited durability under high-voltage cycling, reflecting a fundamental constraint imposed by unaddressed electronic instability.

To bridge this gap, a regeneration strategy that directly intervenes at the electronic origin of degradation is required. In this context, we advance a physical framework based on adsorption-induced bond weakening, whereby controlled adsorption at degraded interfaces modulates local metal-oxygen bonding through electronic interactions rather than macroscopic structural reconstruction. Within this framework, ligand adsorption assumes a fundamentally different role from that in conventional regeneration chemistry. Specifically, acetylacetonate (acac) is introduced not as a chelating agent for metal extraction, but as an electronic mediator capable of interacting with high-valence cobalt centers via *π*-*d* orbital coupling. Such interactions are expected to redistribute Co–O bonding characteristics and alleviate interfacial electronic rigidity, thereby creating a more electronically compliant surface environment. Consequently, the regenerated LCO (R-LCO-Al) exhibits near-ideal stoichiometry, reduced oxygen vacancies, and restored structural ordering, delivering a high specific capacity (179.94 mAh g^−1^ at 0.5 C in 3.0–4.5 V) and 87.0% capacity retention after 200 cycles, which rivals commercial LCO. This conceptual shift positions ligand-mediated electronic regulation as a guiding principle for cathode regeneration, offering a pathway to address electronic-structure instability that has remained largely inaccessible to existing approaches.

## RESULTS

**Figure 1.**
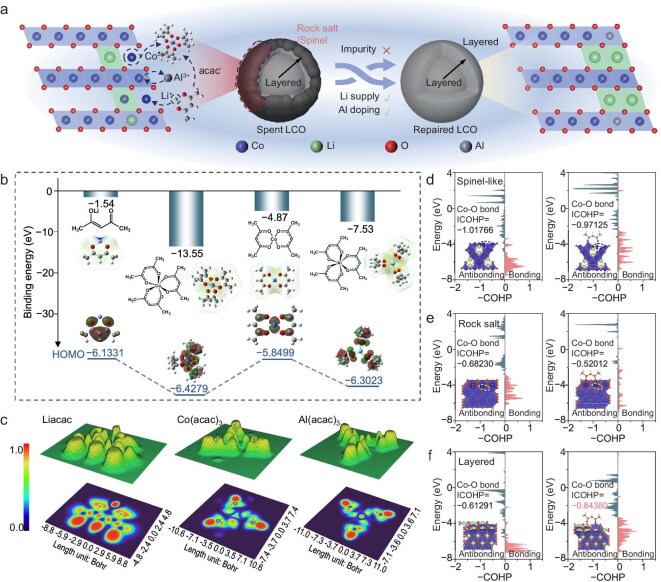
Schematic and mechanism of ligand-induced electronic structure modulation. (a) Schematic illustration of the liquid-phase regeneration mechanism. (b) DFT-calculated binding energy, electrostatic potential, and HOMO energy level of the acetylacetonate chelation products. (c) ELFs of Liacac, Co(acac)_3_ and Al(acac)_3_. Lower ELF values refer to delocalized states and higher ELF values refer to localized states. COHP of Co–O bond in (d) spinel-like phase, (e) rock-salt phase and (f) layered phase with (right) and without (left) acac.

As illustrated in Fig. 1a and Fig. S1, the role of the lithium-aluminum acetylacetonate complex (LAAE) system in the regeneration of S-LCO is schematically summarized. In solution, the acac ligand exists in a metastable coordination environment, where it interacts electronically with surface cobalt species rather than acting solely as a conventional extractant. Through *π-d* orbital interactions, acac perturbs the local Co–O bonding configuration, while preferentially coordinating Co with surface impurity phases, such as spinel and rock-salt phases[24]. Meanwhile, Li^+^ and Al^3+^ ions incorporated within the LAAE framework provide complementary ionic species that can occupy Li sites and vacant Co sites, thereby supporting interfacial reintegration during subsequent thermal treatment.

To clarify the electronic origin of the acac-mediated interaction, density functional theory (DFT) calculations were performed on a series of metal acetylacetonate complexes, M(acac)*_x_* (M = Li^+^, Co^3+^, Co^2+^, Al^3+^), as shown in Fig. 1b. Electrostatic potential mapping reveals a pronounced negative potential localized at the carbonyl oxygen atoms of the acac, indicative of strong electron-donating capability. Correspondingly, the calculated binding energy for Co(acac)_3_ (−13.55 eV) is substantially more negative than those of Liacac (−1.54 eV) and Al(acac)_3_ (−7.53 eV), indicating a strong thermodynamic preference for coordination with Co^3+^. Analysis of the frontier molecular orbitals further shows that the highest occupied molecular orbital (HOMO) of Co(acac)_3_ is localized across both the Co 3*d* orbitals and the *π* system of the acac ligand, reflecting pronounced *d-π* conjugation and the formation of a covalent coordination bond characterized by *π* back-donation.

The nature of metal-ligand bonding was further examined using electron localization function (ELF) analysis (Fig. 1c) [25]. For all complexes, high ELF values are observed around the C=O groups, consistent with the delocalized *π* electrons of the β-diketone framework. In contrast, the ELF characteristics within the metal-oxygen coordination regions (M–O, M = Li, Co, Al) differ markedly. In Liacac, the Li–O region shows low ELF values, indicating dominant electrostatic attraction. By comparison, Co(acac)_3_ exhibits pronounced ELF localization in the Co–O region, consistent with strong covalent bonding, whereas Al(acac)_3_ demonstrates intermediate behavior dominated by ionic character. These results indicate that acac engages in a fundamentally stronger and more covalent interaction with Co^3+^ compared to Li^+^, rationalizing its preferential coordination toward inactive Co species on the S-LCO surface without significant depletion of electrochemically active Li. Notably, when Li^+^ and Co^3+^ coexist, acac ligands can spontaneously assemble into a one-dimensional chain-like bridged precursor, LiCo_1–_*_x_*(acac)_3–2_*_x_*, which is confirmed by Fourier transform infrared spectroscopy and scanning electron microscopy (SEM) (Figs S2 and S3).

Furthermore, the regulatory effect of acac on Co–O bonding within different degraded LCO phases was quantitatively evaluated using crystal orbital Hamilton population (COHP) analysis (Fig. 1d–f). The –COHP profiles indicate that bonding states dominate below the Fermi level, whereas antibonding states are primarily located above it. The integrated COHP (ICOHP) values at the Fermi level provide a quantitative measure of Co–O bonding strength[26]. Upon acac adsorption, the ICOHP values of the spinel and rock-salt phases shift toward less negative values from −1.01766 and −0.68230 to −0.97125 and −0.52012 respectively, indicating reduced bonding-state occupancy and weakened Co–O interactions. In contrast, the layered LCO phase exhibits an opposite trend, which can be attributed to the presence of Li^+^ as an effective charge buffer. The electrostatic interaction between Li^+^ and acac mitigates the direct electron donation to Co^3+^, preventing the population of Co–O antibonding states and thereby preserving structural stability. Collectively, these results demonstrate that acac selectively destabilizes impurity phases at the degraded LCO surface while maintaining the integrity of the layered lattice, effectively softening the rigid electronic interface without invoking bulk lattice disruption.

**Figure 2.**
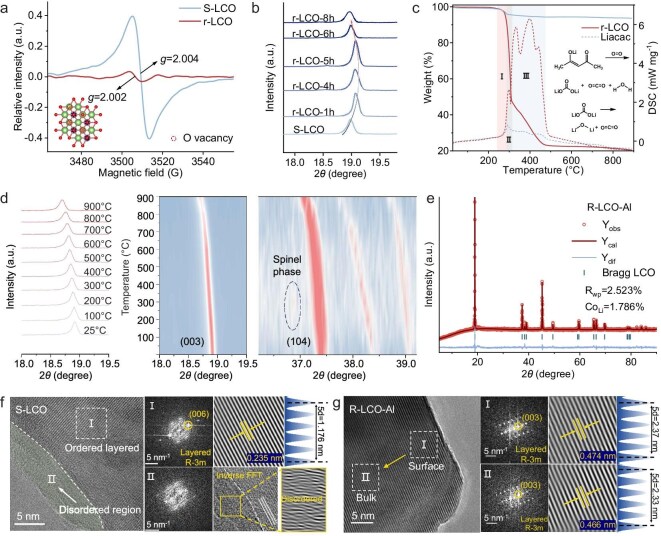
Regeneration process and characterization. (a) EPR spectra of S-LCO and r-LCO. (b) Evolution of XRD patterns of r-LCO as a function of liquid-phase reaction time. (c) TG-DSC curves of the sintering process. (d) *In-situ* temperature-dependent XRD patterns of R-LCO-Al during sintering. (e) Powder XRD pattern and Rietveld refinement results of R-LCO-Al. TEM images and corresponding FFT patterns of (f) S-LCO and (g) R-LCO-Al.

The sample subjected to LAAE treatment is designated r-LCO. To elucidate the surface chemical evolution induced by electronic structure modulation, the regeneration process was systematically examined across the liquid-phase treatment and subsequent annealing process. Electron paramagnetic resonance (EPR) spectroscopy was first employed to probe the evolution of oxygen vacancies (Fig. 2a). The O 1*s* X-ray photoelectron spectroscopy (XPS) spectra further support the presence of undercoordinated oxygen species in degraded LCO (Fig. S4). Pristine S-LCO exhibits a distinct paramagnetic signal at *g* = 2.004, characteristic of electrons trapped at oxygen vacancy sites generated during cycling [27]. Following LAAE treatment, this EPR signal is markedly suppressed in r-LCO, which indicates that the LAAE treatment effectively passivates the paramagnetic centers associated with oxygen vacancies. This primarily originates from the acac-coordination-induced local electronic restructuring and may be further accompanied by the replenishment of lattice oxygen during the subsequent thermal treatment. The strong coordination between the acac ligand and cobalt species promotes local reorganization of Co–O bonding, constituting an electronic-level repair that alleviates interfacial rigidity and primes the surface for ion transport. Consistent with this interpretation, deconvolution of the Co 2*p* XPS spectra (Fig. S5) reveals a marked increase in the relative contribution of the Co^3+^ species in r-LCO compared to S-LCO. The emergence of this higher-valence state is indicative of acac-mediated coordination, plausibly involving transient Co(acac)_3_ formation, which underpins the observed electronic softening and facilitates subsequent ionic rearrangement. The structural response of LCO to liquid-phase treatment was further investigated by X-ray diffraction (XRD). As shown in Fig. 2b, the (003) reflection exhibits a non-monotonic shift, first moving to higher angles and subsequently shifting toward lower angles. The initial rightward shift is attributed to the selective coordination and removal of structurally unstable Co species by acac ligands, accompanied by compensatory relaxation of surrounding O^2−^ anions and slight contraction of the transition metal (TM) layer spacing. The subsequent leftward shift reflects Al^3+^ incorporation into Co sites, where the enhanced covalency of Al–O bonding increases interlayer electrostatic repulsion and expands the *c*-axis lattice parameter. This sequential evolution highlights the dynamic modulation of the near-surface structure enabled by the LAAE system. Notably, this process pre-distributes Al^3+^ at defect sites, thereby providing favorable conditions for its more efficient and uniform diffusion during the subsequent thermal treatment.

The crystallographic reconstruction during annealing was clarified using thermogravimetric-differential scanning calorimetry (TG-DSC) in conjunction with *in-situ* high-temperature XRD (Fig. 2c and d). The decomposition of Liacac proceeds through dehydration, rapid dehydrogenation between 280 and 310°C, and further decomposition associated with Li_2_CO_3_ breakdown at higher temperatures of 310–470°C. Correspondingly, *in-situ* XRD shows a continuous shift of the (003) peak to lower angles upon heating, consistent with progressive lithiation and *c*-axis expansion. Concurrently, diffraction features associated with Co_3_O_4_ spinel impurities gradually weaken to a trace level, indicating near-complete reconstruction toward a well-ordered layered *R*-3*m* structure. Furthermore, we also explore the applicability of LAAE to Ni-rich NCM cathodes. However, the excessively high reactivity of Ni led to severe dissolution, causing structural collapse and surface wrinkling (Fig. S6), highlighting the specificity of the LAAE system for LCO repair.

The annealed product is designated as R-LCO-Al. Rietveld refinement confirms a dramatic reduction in the Co/Li anti-site defect to 1.786% and the reduction of Co_3_O_4_ phase impurities to a trace level (Fig. 2e and Fig. S7, Tables S1–S3), aligning perfectly with the *in-situ* observations. High-resolution transmission electron microscopy further corroborates the restoration of crystallographic order of R-LCO-Al (Fig. 2f and g, Fig. S8). Whereas S-LCO exhibits near-surface short-range disorder with mixed spinel and rock-salt domains, R-LCO-Al displays a uniformly ordered layered structure with sharp (003) reflections in the fast Fourier transform patterns. A modest expansion of the interplanar spacing is observed near the surface relative to the bulk, consistent with preferential Al^3+^ incorporation facilitated by the electronically softened interface. Collectively, these results demonstrate that the LAAE strategy enables a transition from a rigid, electronically degraded interface to a softened, reconfigurable one through the coupling of liquid-phase electronic structure modulation and solid-state annealing. This synergistic process repairs surface defects, restores lattice order, and stabilizes the layered framework, thereby establishing a mechanistic foundation for high-performance LCO regeneration.

**Figure 3.**
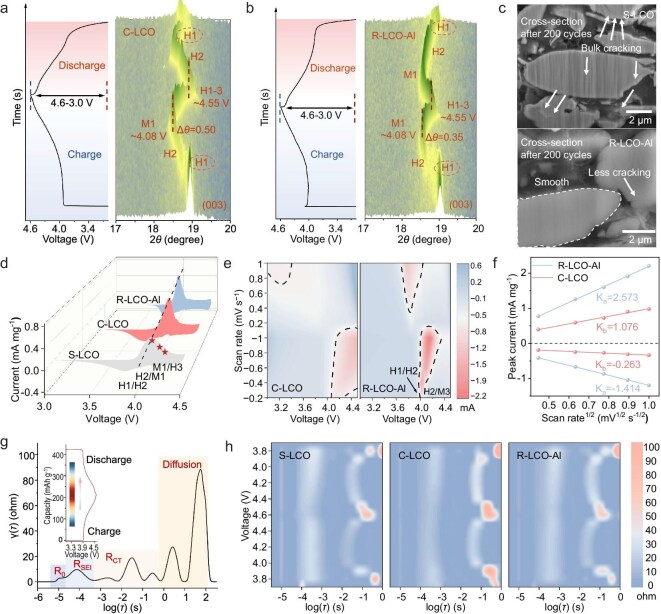
Structural evolution and electrochemical kinetic validation. *In-situ* XRD patterns of (a) C-LCO and (b) R-LCO-Al during cycling at 4.6 V (vs. Li^+^/Li). (c) FIB-SEM cross-sectional images of S-LCO and R-LCO-Al after 200 cycles. (d) CV curves of various LCO samples at a scan rate of 0.1 mV s^−1^. (e and f) CV contour maps of C-LCO and R-LCO-Al at different scan rates, and the linear relationships between peak current and square root of scan rate. (g and h) Electrochemical impedance spectroscopy (EIS) and corresponding DRT analysis for different LCO.

The restoration efficacy of the LAAE regeneration strategy was comprehensively evaluated by probing the lattice dynamics of R-LCO-Al under high-voltage operation. Using *in-situ* XRD at 4.6 V vs. Li^+^/Li, the structural evolution of R-LCO-Al was directly compared with that of commercial LCO (C-LCO) (Fig. 3a and b). Upon Li^+^ extraction, layered LCO undergoes a series of phase transitions (H1→H2→M1→H3→H1-3→O1), among which the H1-3 and O1 transformations are the primary origins of irreversible capacity degradation due to pronounced anisotropic lattice strain and irreversible oxygen release [28]. At voltages exceeding 4.5 V, collective sliding of CoO_2_ slabs induces a sharp contraction of the *c*-axis spacing, amplifying structural irreversibility [29]. In this context, R-LCO-Al exhibits a substantially smaller shift of the (003) reflection during charging, indicative of lowered *c*-axis strain. This stabilization is attributed to Al^3+^ incorporation, which reinforces the layered framework by strengthening the ionic component of Co–O bonding, thereby delaying the onset of detrimental high-voltage phase transitions. The impact of structural stabilization on cycling-induced mechanical degradation was visualized by focused ion beam scanning electron microscopy (FIB-SEM) cross-sectional analysis (Fig. 3c). After prolonged cycling, the S-LCO electrode displays extensive intergranular cracking, pervasive porosity, and internal microfractures, which facilitate electrolyte penetration and intensify parasitic interfacial reactions. Such defects concentrate mechanical stress during repeated lithiation and delithiation, accelerating capacity fade and structural degradation [30]. In contrast, R-LCO-Al maintains a dense and intact microstructure after 200 cycles, with no evidence of catastrophic grain fracture. The result stands in sharp contrast to the particle morphology and lithium loss of S-LCO (Figs S9–S11). Dynamic EIS monitoring further indicates that the charge transfer resistance of R-LCO-Al increases more slowly during extended cycling, demonstrating significantly superior interfacial stability compared to S-LCO (Fig. S12). This superior morphological stability and kinetic performance originate from the pinning effect of Al^3+^ dopants and the dual benefits of near-stoichiometric lattice repair, which collectively mitigate lattice strain and suppress phase-transition-induced stress accumulation during cycling [31]. The LAAE regeneration strategy not only restores the bulk structure of the material but more importantly reconstructs and stabilizes its electrochemical interface.

Electrochemical kinetics were further investigated to elucidate the consequences of interfacial softening and structural stabilization. Cyclic voltammetry (CV) recorded at 0.1 mV s^−1^ reveals well-defined and sharp redox couples for R-LCO-Al at approximately 3.92, 4.08, and 4.18 V (Fig. 3d), corresponding to the characteristic H1/M/H2, H2/M1, and M1/H3 phase transitions of layered LCO. In contrast, S-LCO exhibits broadened and attenuated peaks, indicating severe polarization and sluggish reaction kinetics. Variable scan-rate CV analysis further elucidates the kinetic advantages of R-LCO-Al (Fig. 3e and f), which demonstrates more symmetric peak profiles and narrowed full-width at half-maximum, underscoring diminished kinetic hysteresis and enhanced reversibility. Analysis based on the Randles–Ševčík equation yields substantially larger slopes for R-LCO-Al compared with C-LCO, indicating a substantially higher apparent Li^+^ diffusion coefficient. This improvement is attributed to the synergistic effects of electronically softened interfaces created by LAAE treatment and Al^3+^-induced *c*-axis expansion, which collectively lower the activation barrier for Li^+^ mobility. Consistently, galvanostatic intermittent titration technique measurements reveal lower polarization over the entire voltage window (Fig. S13).

To further resolve the interfacial charge-transfer dynamics, *in-situ* electrochemical impedance spectroscopy (EIS) coupled with distribution of relaxation times (DRT) analysis was employed (Fig. 3g and h). Unlike conventional equivalent circuit fitting, DRT analysis enables separation of overlapping impedance contributions within the 3.0–4.5 V range into ohmic resistance, solid electrolyte interphase (SEI) resistance, charge-transfer resistance, and electrolyte diffusion resistance [32,33]. R-LCO-Al exhibits a pronounced reduction in both SEI-related and charge-transfer relaxation features, accompanied by a more centralized and symmetric relaxation time distribution. These characteristics signify a homogenized electrode/electrolyte interface with facilitated charge transfer and accelerated ion transport, culminating in a kinetic basis for the superior high-voltage electrochemical performance of the regenerated cathode.

**Figure 4.**
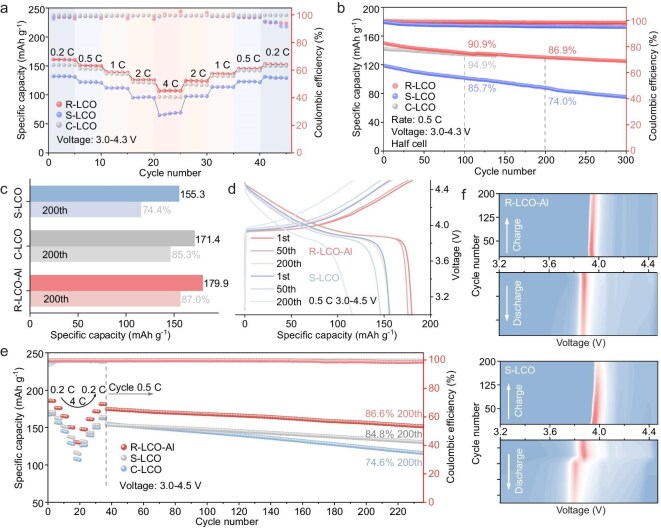
Electrochemical performance evaluation of the regenerated materials. (a) Rate capability of S-LCO, C-LCO, and R-LCO in the voltage range of 3.0–4.3 V. (b) Cycling stability of S-LCO, C-LCO, and R-LCO at 0.5 C within 3.0–4.3 V. (c) Discharge specific capacities of S-LCO, C-LCO, and R-LCO-Al before and after 200 cycles at 0.5 C (3.0–4.5 V). (d) Charge/discharge profiles of S-LCO and R-LCO-Al at 0.5 C between 3.0 and 4.5 V. (e) Rate capability and long-term cycling performance of S-LCO, C-LCO, and R-LCO-Al at 0.5 C in the voltage window of 3.0–4.5 V. (f) d*Q*/d*V* contour maps of S-LCO and R-LCO-Al under the same conditions.

The regenerative efficacy of the LAAE strategy was further assessed by systematically comparing the rate capability and cycling stability of the repaired cathodes across different voltage windows. As shown in Fig. 4a, the LAAE-treated sample with only lithium compensation (R-LCO) delivers specific capacities of 161.24, 151.12, 139.55, 125.92, and 106.88 mAh g^−1^ at rates of 0.2, 0.5, 1, 2, and 4 C, respectively, within 3.0–4.3 V window. These values are comparable to those of pristine C-LCO and significantly superior to those of degraded S-LCO, demonstrating effective restoration of Li^+^ content and transport kinetics. Despite its recovered rate performance, R-LCO exhibits limited cycling durability. When cycled at 0.5 C, it retains only 90.9% of its capacity after 100 cycles, which is inferior to that of C-LCO (94.9%) under identical conditions (Fig. 4b). This disparity underscores an intrinsic limitation of relithiation-only regeneration. Although Liacac-mediated relithiation replenishes the bulk Li inventory, it fails to stabilize the TM framework compromised by Co dissolution. As a consequence, structural degradation persists during prolonged cycling, underscoring the necessity of an additional stabilizing agent beyond mere Li compensation.

The incorporation of Al^3+^ within LAAE system fundamentally alters this outcome. As illustrated in Fig. 4c–e, the regenerated R-LCO-Al cathode exhibits exceptional stability under high-voltage cycling between 3.0 and 4.5 V. An initial discharge capacity of 179.94 mAh g^−1^ is achieved at 0.5 C, with 87.0% of this capacity retained after 200 cycles. In contrast, S-LCO suffers rapid performance decay, with discharge capacities declining to 115.82 mAh g^−1^. To further evaluate the practical potential of this regeneration strategy, we fabricated a high-loading R-LCO-Al electrode and tested its electrochemical performance (Fig. S14). This electrode exhibits excellent long-term cycling stability at 0.5 C, delivering an initial specific capacity of 173.18 mAh g^−1^ and retaining 95.16% of its capacity after 100 cycles. Insight into the electrochemical reversibility is provided by the differential capacity (d*Q*/d*V*) profiles (Fig. 4f). R-LCO-Al displays well-defined and sharp oxidation and reduction peaks centered at ∼3.92 and 3.87 V, corresponding to the characteristic phase transitions of LCO. Notably, these features remain nearly unchanged after extended cycling, whereas S-LCO shows substantial peak broadening and voltage shifts. The minimal evolution of the d*Q*/d*V* profiles for R-LCO-Al indicates highly reversible phase behavior and suppressed structural degradation. The competitive performance of R-LCO-Al is further contextualized through comparison with representative regeneration strategies reported in the literature (Table S4). Together, these results demonstrate that the multifunctional LAAE strategy, combining Li replenishment with Al^3+^-mediated framework stabilization, enables sustained electrochemical performance in regenerated LCO cathodes under aggressive cycling conditions.

**Figure 5.**
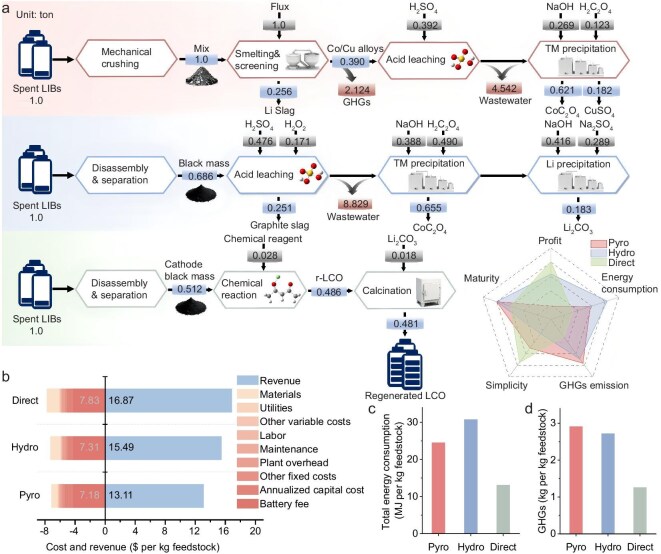
Techno-economic analysis. (a) Material flow of the Pyro, Hydro, and Direct processes for 1.0 t spent batteries. (b) Cost and revenue analysis. (c) Total energy consumption. (d) GHGs emissions analysis.

A holistic assessment of the industrial feasibility of the proposed DR strategy necessitates a quantitative comparison of its techno-economic and environmental performance against established pyrometallurgical (Pyro) and hydrometallurgical (Hydro) recycling routes. To ensure consistency, all processes were evaluated on the basis of treating 1 metric ton of spent lithium-ion battery cells, with the corresponding material inputs provided in Table S5. The fundamental distinctions among these recycling strategies are illustrated through material flow analysis (Fig. 5a). In the Pyro route, shredded battery materials are subjected to high-temperature smelting (>1400°C), reducing TMs to metallic alloys or simple compounds, followed by downstream conversion into products such as CoC_2_O_4_ and CuSO_4_. This process is inherently energy-intensive and time-consuming, with Li often lost to slag formation or volatilization. In contrast, the Hydro route relies on multistep acid leaching, solvent extraction, and precipitation to recover metals as purified salts, including Li_2_CO_3_, CoC_2_O_4_, and CuSO_4_. While effective in elemental separation, this process incurs substantial chemical consumption and generates large volumes of wastewater, imposing significant environmental management burdens. The economic implications of these process differences are reflected in the cost and revenue analysis (Fig. 5b, Tables S6 and S7). Although the use of LAAE reagents introduces a direct raw-material cost in the DR process, the elimination of energy-intensive smelting, extensive chemical separation, and complex waste treatment substantially reduces overall operating expenses. More importantly, DR yields a directly reusable cathode material of R-LCO-Al, which possesses a considerably higher market value than commodity metal salts. As a result, the estimated net profits of the Pyro, Hydro, and DR routes were 5.93, 8.18, and 9.04 US$ kg^−1^ feedstock, respectively (Fig. S15), underscoring the superior profitability of the DR approach. Beyond economic considerations, the environmental merits of DR are particularly pronounced. A cradle-to-gate lifecycle assessment was conducted to quantify cumulative energy demand and associated greenhouse gas (GHG) emissions (Fig. 5c and d). The Pyro process exhibits a high carbon footprint owing to its extreme thermal requirements, while the Hydro route carries substantial indirect emissions linked to chemical production and wastewater treatment. In contrast, the DR strategy operates under comparatively mild conditions and avoids extensive chemical processing, resulting in a reduction of total energy consumption by ∼40%–60% compared to conventional routes. This decrease translates directly into a commensurate reduction in associated GHG emissions. Collectively, these results indicate that the DR strategy not merely offers a viable economic pathway but also represents a low-carbon recycling framework aligned with broader climate mitigation objectives.

## CONCLUSION

In summary, this work directly addresses the central premise raised at the outset of this study: that the irreversible degradation of LiCoO_2_ cathodes originates from electronic structure destabilization rather than from isolated compositional loss or structural disorder. We demonstrate that this electronic instability, manifested as rigidified Co–O bonding and interfacial electron localization, can be reversibly regulated at the molecular level through the LAAE strategy. By introducing acac ligands as electronic mediators rather than conventional chelating agents, the LAAE system enables adsorption-induced *π*→*d* electron donation to cobalt centers, selectively weakening Co–O covalency at degraded surfaces and transforming the electronically rigid interface into a softened, reconfigurable state. This electronic reconditioning facilitates the removal of inactive cobalt species, the healing of oxygen vacancies, and the controlled incorporation of Al^3+^, collectively restoring the structural-electronic coherence of the layered oxide framework. By re-establishing electronic compatibility prior to compositional repair, the regenerated LiCoO_2_ recovers electrochemical behavior comparable to commercial materials under high-voltage operation. As a consequence, the regenerated material (R-LCO-Al) delivers exceptional electrochemical performance, including a high specific capacity (179.94 mAh g^−1^ at 0.5 C within 3.0–4.5 V) and outstanding cycling stability (87.0% capacity retention after 200 cycles). More broadly, this work establishes electronic structure modulation via ligand adsorption as a generalizable regeneration approach, offering a low-energy, chemically efficient route for sustainable battery recycling and for the design of future molecular media capable of regulating complex oxide interfaces.

## METHODS

Detailed methods can be found in the online supplementary information.

## Supplementary Material

nwag369_Supplemental_File
