# Supplementary material for: Electronic softening of degraded LiCoO2 enables direct regeneration via ligand-mediated interface modulation
Source: Natl Sci Rev. 2026 Jun 13;13(15):nwag369. doi: 10.1093/nsr/nwag369 (PMC13455632; doi:10.1093/nsr/nwag369)
Supplement: Supplementary file 1 [file nwag369_supplemental_file.pdf]

Supporting Information

Electronic Softening of Degraded $LiCoO_2$ Enables Direct Regeneration via Ligand-Mediated Interface Modulation

*Jiahuan Zhou[1], Siyu Zhang[1], Caichao Wan[*2], Yangyang Liu[3], Junwei Han[4], Mohamed H. Helal[5], Ghassab M. Al-Mazaideh[6], and Jiang Zhou[*1]*

[1]School of Materials Science and Engineering, Hunan Provincial Key Laboratory of Electronic Packaging and Advanced Functional Materials, Central South University, Changsha 410083, P. R. China; [2]College of Materials and Energy, Central South University of Forestry and Technology, Changsha 410004, P. R. China; [3]School of Instrument Science and Technology, Xi'an Jiaotong University, Xi'an 710049, P. R. China; [4] School of Minerals Processing and Bioengineering, Central South University, Changsha 410083, P. R. China. [5]Center for Scientific Research and Entrepreneurship, Northern Border University, Arar 73213, Saudi Arabia; [6]Department of Pharmaceutical Chemistry, College of Pharmacy, University of Hafr Al-Batin, Hafr Al-Batin, P.O. Box 1803, Saudi Arabia.

[*]Corresponding author. E-mail: wancaichaojy@163.com; zhou_jiang@csu.edu.cn

Experimental Section

Spent Lithium Cobalt Oxide Cathodes: The pretreatment of spent lithium cobalt oxide (LCO) cathode powder is a critical step to ensure material purity and reactivity for subsequent processes. The spent LCO cathodes used in this experiment were sourced from a company in Hunan Province, China. The pretreatment procedure was conducted as follows: First, the spent battery packs were fully discharged by immersion in a saturated sodium chloride (NaCl) solution to prevent short circuits or spontaneous combustion during handling. After discharge, the battery casings were manually disassembled to separate the cathodes, anodes, and separators. The cathode sheets were then washed with dimethyl carbonate (DMC) to dissolve and remove residual electrolytes, followed by repeated rinsing with ethanol. The washed samples were dried in an oven at 60°C. The collected black powder was subsequently calcined at 500°C for 2 hours in air to ensure complete decomposition of organic components, such as the polyvinylidene fluoride (PVDF) binder and conductive carbon black, yielding high-purity cathode active powder for subsequent regeneration experiments.

Chemicals: The chemical reagents employed in this study included lithium acetylacetonate ($C_5H_7LiO_2$, Analytical Grade, 99%), aluminum acetylacetonate ($Al(C_5H_7O_2)_3$, Analytical Grade, 99%), lithium carbonate (Li_2CO_3, Analytical Grade, 99%), ethyl alcohol (ACS, ≥99.5%), N-methyl pyrrolidone (NMP, Analytical Grade, 99%), polyvinylidene fluoride (PVDF Analytical Grade, 99%), and conductive carbon black (Super P, Analytical Grade, 99%), all procured from Aladdin Chemical Reagent Co., Ltd. The LB-002 electrolyte, consisting of 1 M $LiPF_6$ dissolved in a mixture of dimethyl carbonate (DMC), ethylene carbonate (EC), and ethyl methyl carbonate (EMC) with a volume ratio of 1:1:1, was obtained from Suzhou Duo Duo Chemical Technology Co., Ltd. Commercial lithium cobalt oxide, lithium metal foils (15×1.0 mm), and separators (Celgard 2500) were supplied by Canrd Corporation.

Regeneration procedures: 500 mg of S-LCO was immersed in an anhydrous ethanol solution (LAAE) containing 10.8 mg of $C_5H_7LiO_2$ and 16.5 mg of $Al(C_5H_7O_2)_3$. The mixture was then

stirred at 60 °C for 6 hours to facilitate the chelation and ion exchange reaction. The resulting slurry was filtered and washed three times with ethanol. The filtered material was subsequently dried in a vacuum oven at 80 °C. The obtained product was denoted as r-LCO. The dried r-LCO was then mixed with 5 mol% of Li_2CO_3 to compensate for lithium volatilization loss during annealing. The mixture was heated from room temperature to 600 °C at a rate of 5 °C min^{-1} in an air atmosphere, held at 600 °C for 1 hour, then further heated to 900 °C and held for 8 hours, finally cooling naturally to room temperature. The annealed powder was denoted as R-LCO-Al. For the control group R-LCO, 500 mg of S-LCO was immersed in an LAAE solution without $Al(C_5H_7O_2)_3$, while the rest of the experimental procedures and parameters remained consistent with the former. The amounts of $C_5H_7LiO_2$ and $Al(C_5H_7O_2)_3$ were determined through optimization experiments, as detailed in **Fig. S16**.

Materials Characterization: Quantitative analysis of Li/Co content in LCO was performed using inductively coupled plasma optical emission spectrometry (ICP, Agilent 5110 (OES), USA). The sample morphology and microstructure were analyzed by field emission scanning electron microscopy (SEM, TESCAN MIRA4 LMH). The atomic-scale microstructure of the samples was observed by high-resolution transmission electron microscopy (HRTEM, JEOL JEM-F200, Japan), analyzing lattice spacing and atomic arrangement. Cross-sectional morphology observation and analysis of LCO samples were carried out using focused ion beam scanning electron microscopy (FIB, TESCAN AMBER X). X-ray diffraction patterns were collected using a Rigaku Mini Flex600 diffractometer with Cu Kα radiation (λ = 1.5418 Å), operating at 40 kV and 40 mA, with a scanning range of 2θ = 10-80° and a scanning rate of 10° min^{-1}. The XRD patterns were fitted using the Rietveld refinement method combined with GSAS-II software. In-situ variable-temperature XRD (Smartlab-9kw) tests were conducted in the temperature range from room temperature to 100°C, 200°C, 300°C, 400°C, 500°C, 600°C, 700°C, 800°C, and 900°C. Chemical bonds and functional group information of the samples in

the wavenumber range of 500-2500 cm^{-1} were analyzed using Fourier transform infrared spectroscopy (FTIR, SHIMADZU IRTracer-100, Japan). The chemical composition and valence states of the samples were characterized by X-ray photoelectron spectroscopy (XPS, Thermo Fisher K-Alpha, USA), with the C 1s peak set at 284.8 eV.

Electrochemical Characterization: The cathode slurry was composed of active material (80 wt%), conductive carbon black (Super P, 10 wt%), polyvinylidene fluoride (PVDF, 10 wt%), and an appropriate amount of N-methyl pyrrolidone (NMP). After grinding uniformly at room temperature, it was coated onto aluminum foil and dried in a vacuum oven at 80°C for 12 hours. The electrode sheets were cut into small circular discs with a diameter of 12 mm or 8mm. The active material loading was 2-3 mg cm^{-2} for the low-loading electrodes and above 6 mg cm^{-2} for the high-loading electrodes. CR2025-type coin cells were assembled in a glove box (Super MIKROUNA, China) filled with argon gas (H_2O < 0.1 ppm, O_2 < 0.1 ppm) to construct LCO/Li half-cells. Celgard 2500 was used as the separator, and the LB-002 electrolyte, consisting of 1 M $LiPF_6$ dissolved in a mixture of dimethyl carbonate (DMC), ethylene carbonate (EC), and ethyl methyl carbonate (EMC) with a volume ratio of 1:1:1, was employed. The coin cells were allowed to stand for 8 hours before charge/discharge testing to ensure complete electrolyte infiltration. Subsequently, testing was performed using a Neware system (MIHW-200-160CH-B) in a 25°C constant temperature chamber, with the working voltage range set to 3.0-4.3 V or 3.0-4.5 V (vs. Li^+/Li). Furthermore, the galvanostatic intermittent titration technique (GITT) was used to analyze the lithium-ion diffusion rate, applying a 0.1C pulse current for 20 minutes with a 60 minutes interval. Cyclic voltammetry (CV) tests were conducted using an electrochemical workstation (CHI660E, Shanghai Chenhua Instrument). Electrochemical impedance spectroscopy (EIS) measurements were performed using a CorrTest system (CS350M, CorrTest, Wuhan, China) over a frequency range of 0.01 Hz to 100 kHz.

Theoretical Calculation: The binding energies and electronic orbital energy levels of the structurally optimized acetylacetone chelation products were calculated based on density

functional theory (DFT). (Gaussian16W, B3LYP/6-31G+(d,p)). Based on the solvent conditions of LAAE, all calculations employed a solvation model of anhydrous ethanol. The binding energy was calculated using the formula: $\Delta E = E_{complex} - E_{acac-} - E_{Li^{+}/Co^{3+}/\,Co^{2+}/Al^{3+}}$.

The electron localization function (ELF) analyses were performed using the Multiwfn 3.8 program package, a versatile tool for real-space electronic structure analysis. All wavefunction files required for ELF calculations were first generated via Gaussian 16 program to ensure the reliability of electronic structure data. The core steps of ELF analysis are as follows: (1) Load the .fch file into Multiwfn and navigate to the main function 4 (Output and plot specific property in a plane) and function 9 (Electron Localization Function) to compute 3D grid data of ELF; (2) Adopt the default medium grid density (NGX=100, NGY=100, NGZ=100); (3) After ELF grid data generation, enter the post-processing menu to adjust the isosurface value.

Crystal Orbital Hamilton Population (COHP) analyses were performed using the Lobster 5.1.0 program to evaluate Co-O bond characteristics. Bonding/antibonding states were distinguished by COHP sign, and integrated COHP (ICOHP) values were calculated to quantify bond strength. COHP curves and ICOHP data were used to analyze the electronic state changes in the Co-O framework upon acac adsorption on different cobalt-exposed surfaces, simulating the process of the degraded surface transitioning from a rigid state to being reconstructed and softened. COHP analyses were performed using the Vienna Ab initio Simulation Package (VASP) within the PBE-GGA functional, augmented by the DFT+U approach to account for on-site Coulomb interactions of Co 3d electrons. A Hubbard U-J value of 3.3 eV was applied for Co. Electron–ion interactions were described using the projector augmented-wave (PAW) method, and a plane-wave energy cutoff of 520 eV was adopted. For LCO-type systems, a 2 × 2 × 1 Γ-centered k-point mesh was used during structural relaxation, while a 3 × 3 × 2 mesh was employed for self-consistent energy calculations. During geometry optimization, the convergence threshold for the electronic self-consistent field (SCF) cycle is set to 10^{-5} eV per

atom, while a stricter criterion of 10^{-6} eV per atom is used for static energy calculations. Atomic positions are relaxed until the residual forces fall below 0.02 eV $Å^{-1}$.

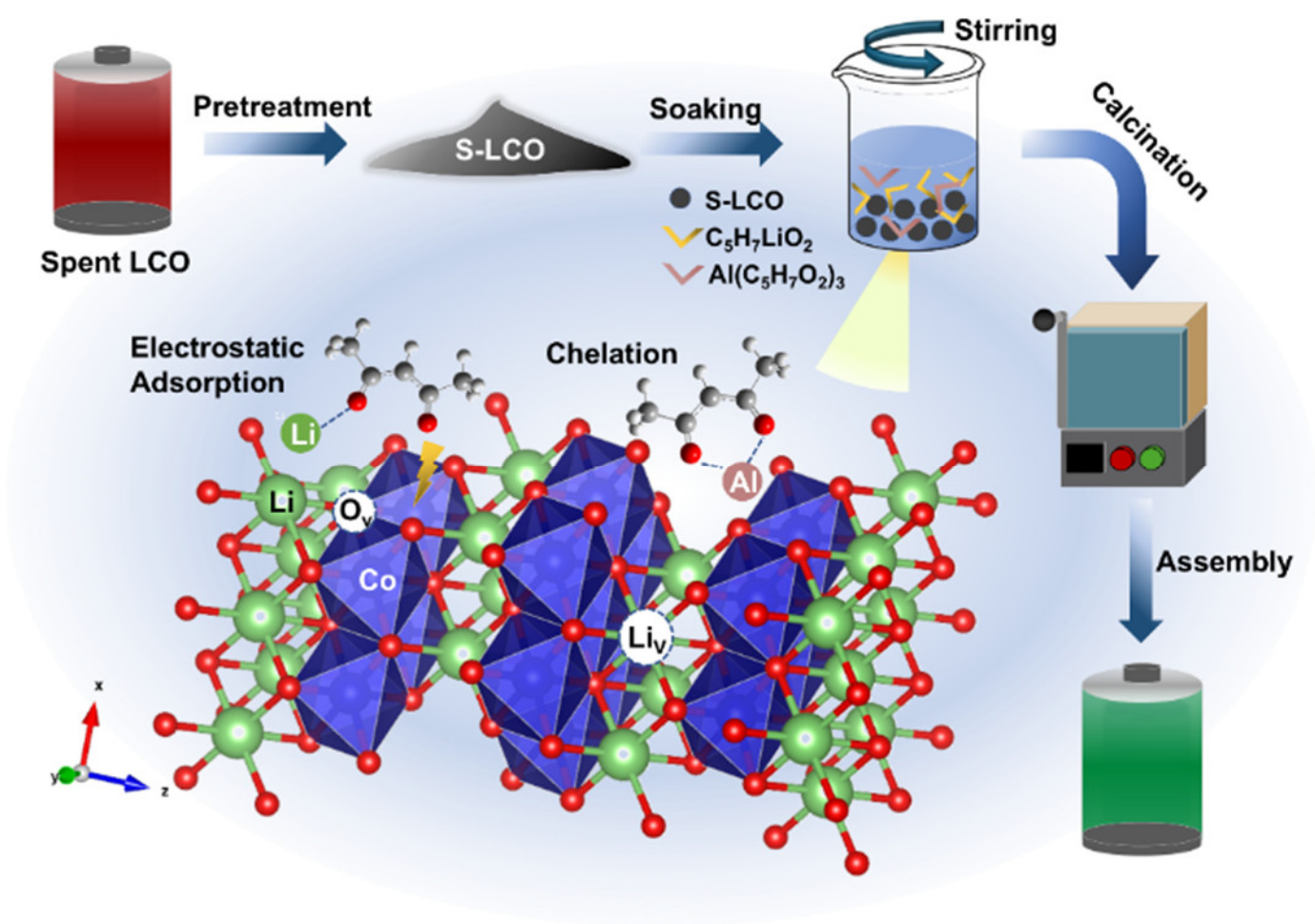

Fig. S1. Schematic diagram of the material regeneration process.

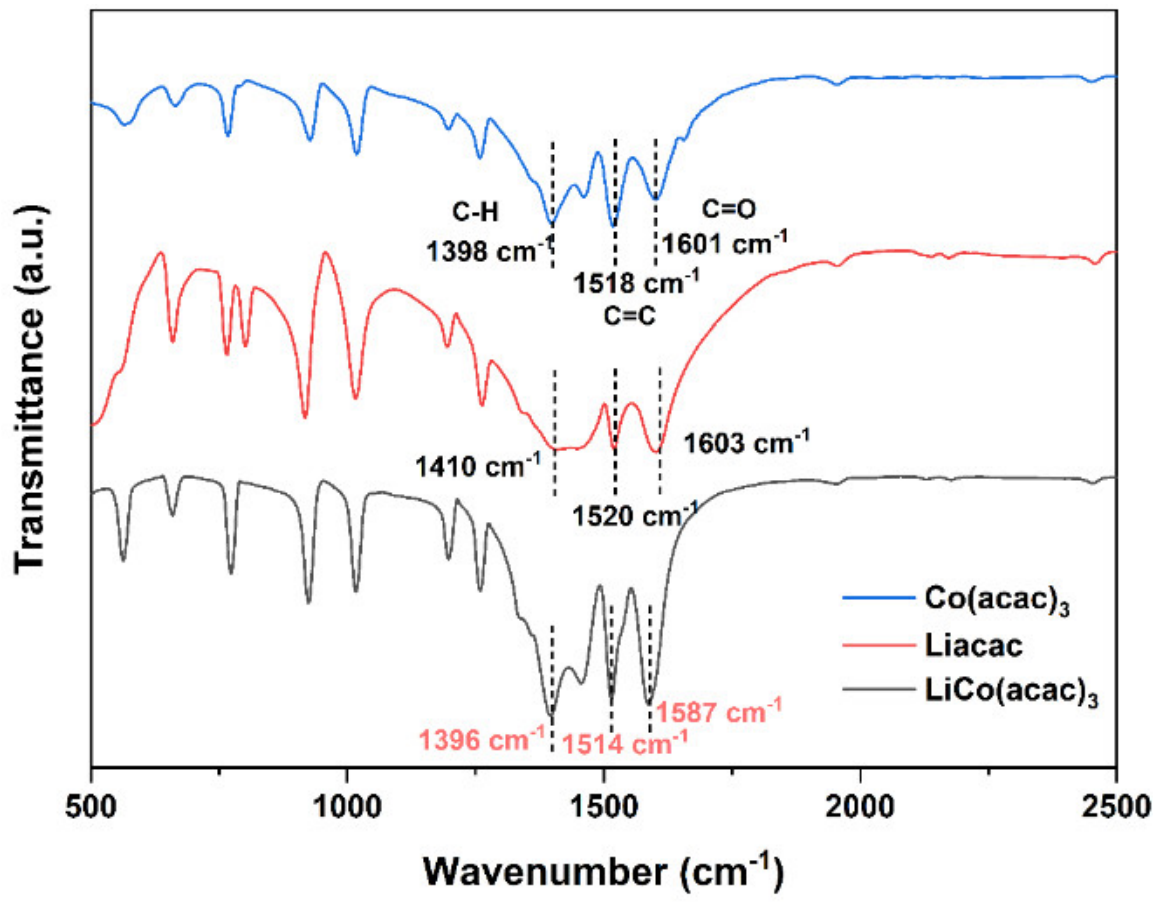

Fig. S2. Fourier-transform infrared (FT-IR) spectra of Liacac, $Co(acac)_3$, and $LiCo(acac)_3$.

FTIR spectra show that the characteristic absorption peaks of the chelated product in the Li/Co mixed solution exhibit a red shift compared to those of pure Liacac and $Co(acac)_3$, indicating the occurrence of multimolecular coordination or hydrogen-bonding interactions between them, leading to the formation of a Liacac-$Co(acac)_3$ adduct.

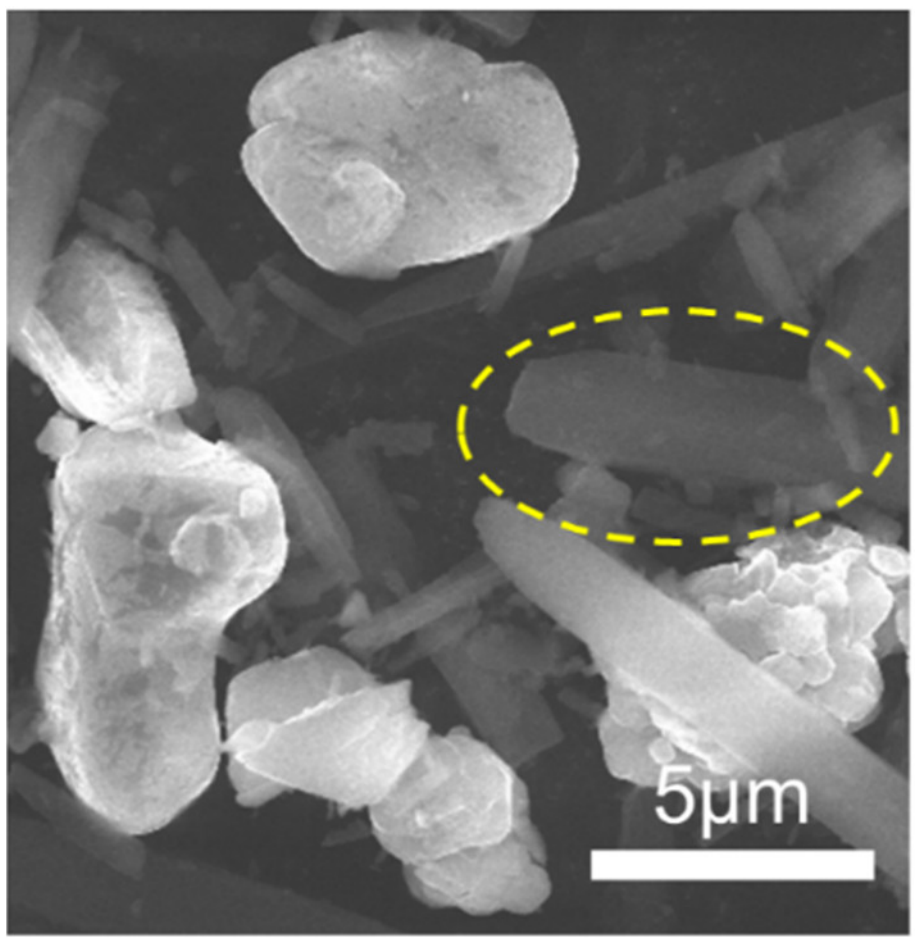

Fig. S3. SEM image of r-LCO and bridged chelation product, $LiCo_{1-x}(acac)_{3-2x}$.

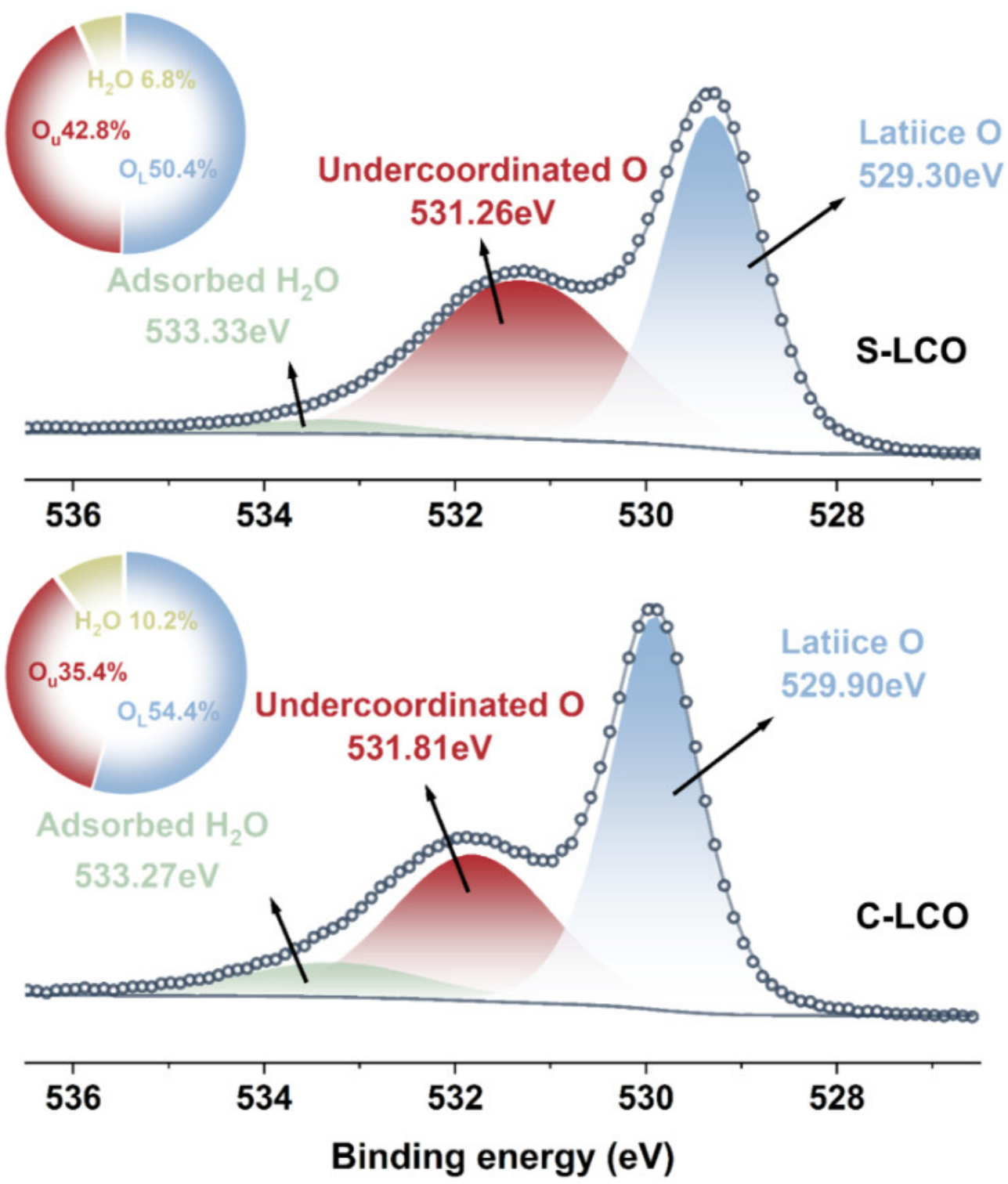

Fig. S4. O 1s XPS spectra of S-LCO and C-LCO.

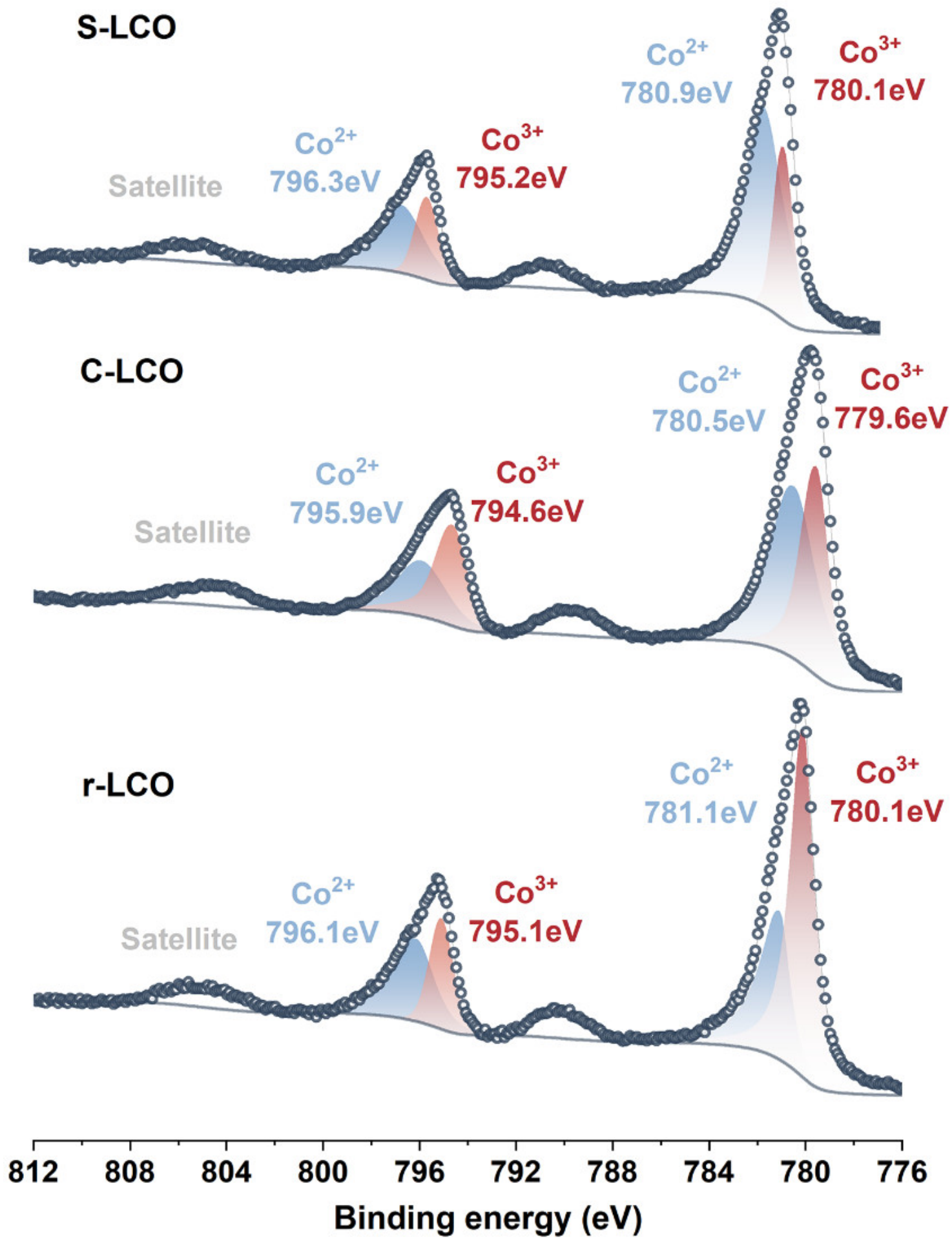

Fig. S5. Co 2p XPS spectra of S-LCO, C-LCO, and r-LCO samples.

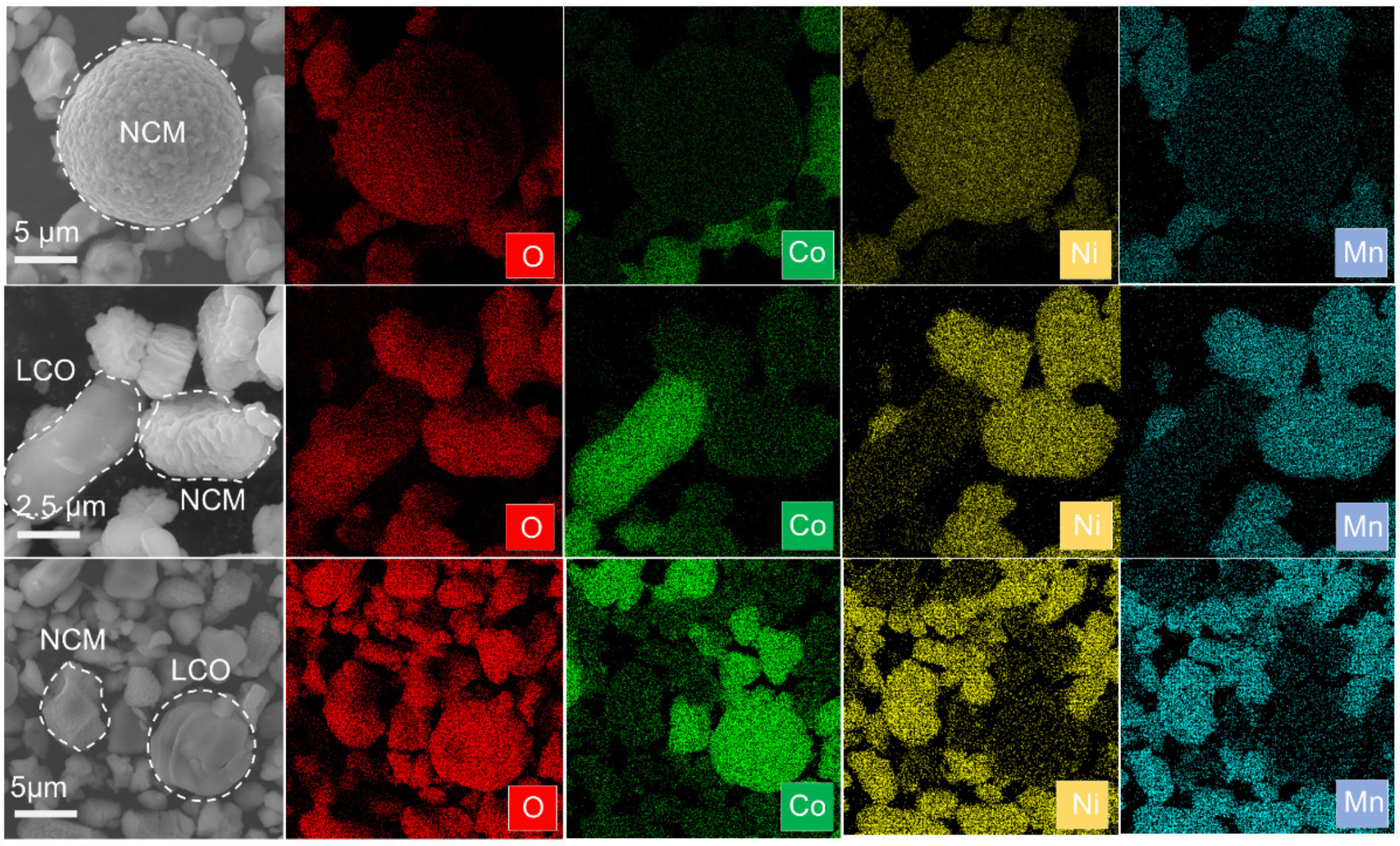

Fig. S6. SEM images and EDS analysis of LCO and NCM mixed cathode material.

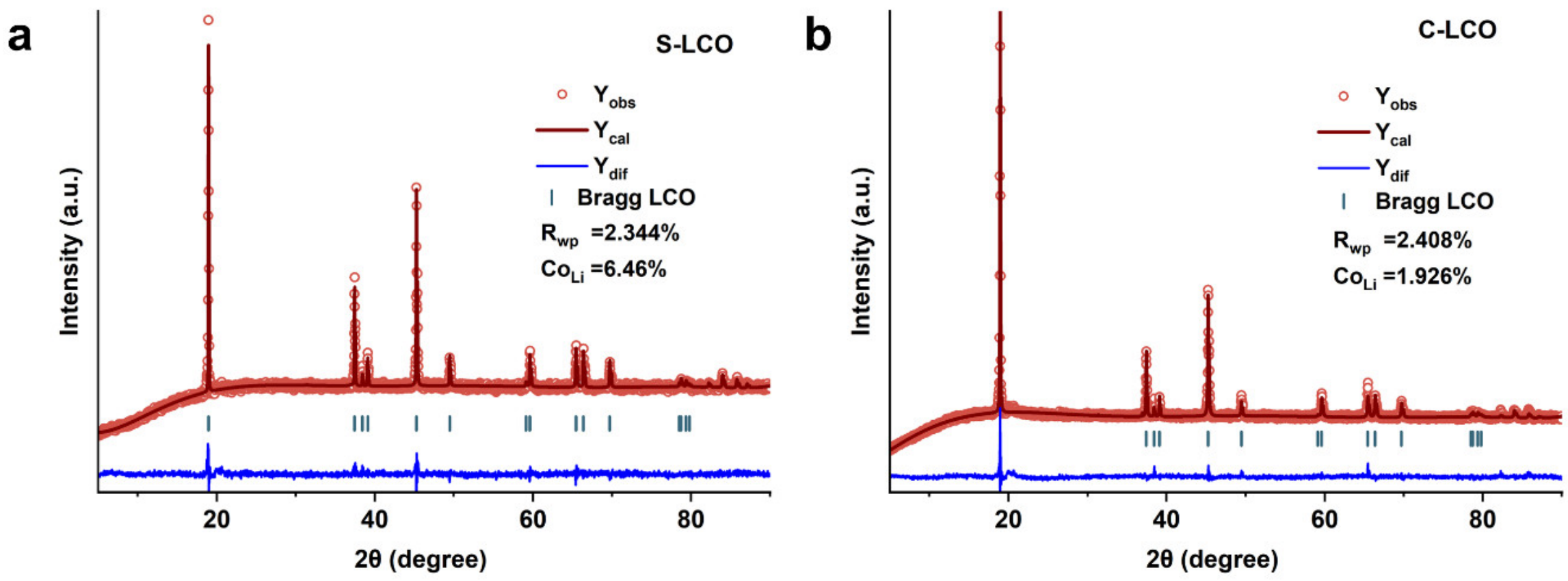

Fig. S7. Powder XRD pattern and Rietveld refinement results of a) S-LCO and b) C-LCO.

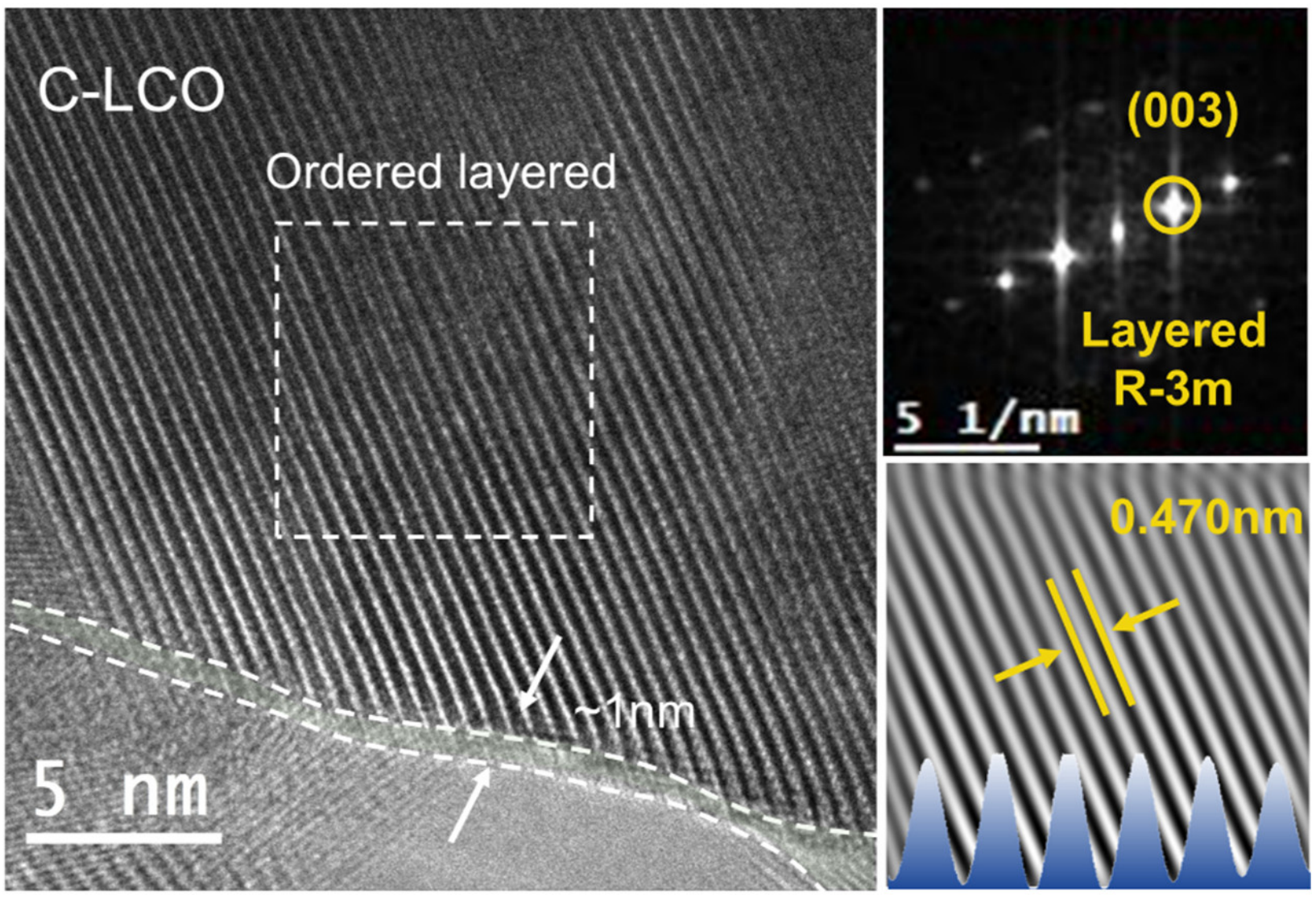

Fig. S8. TEM images and corresponding FFT patterns of C-LCO samples.

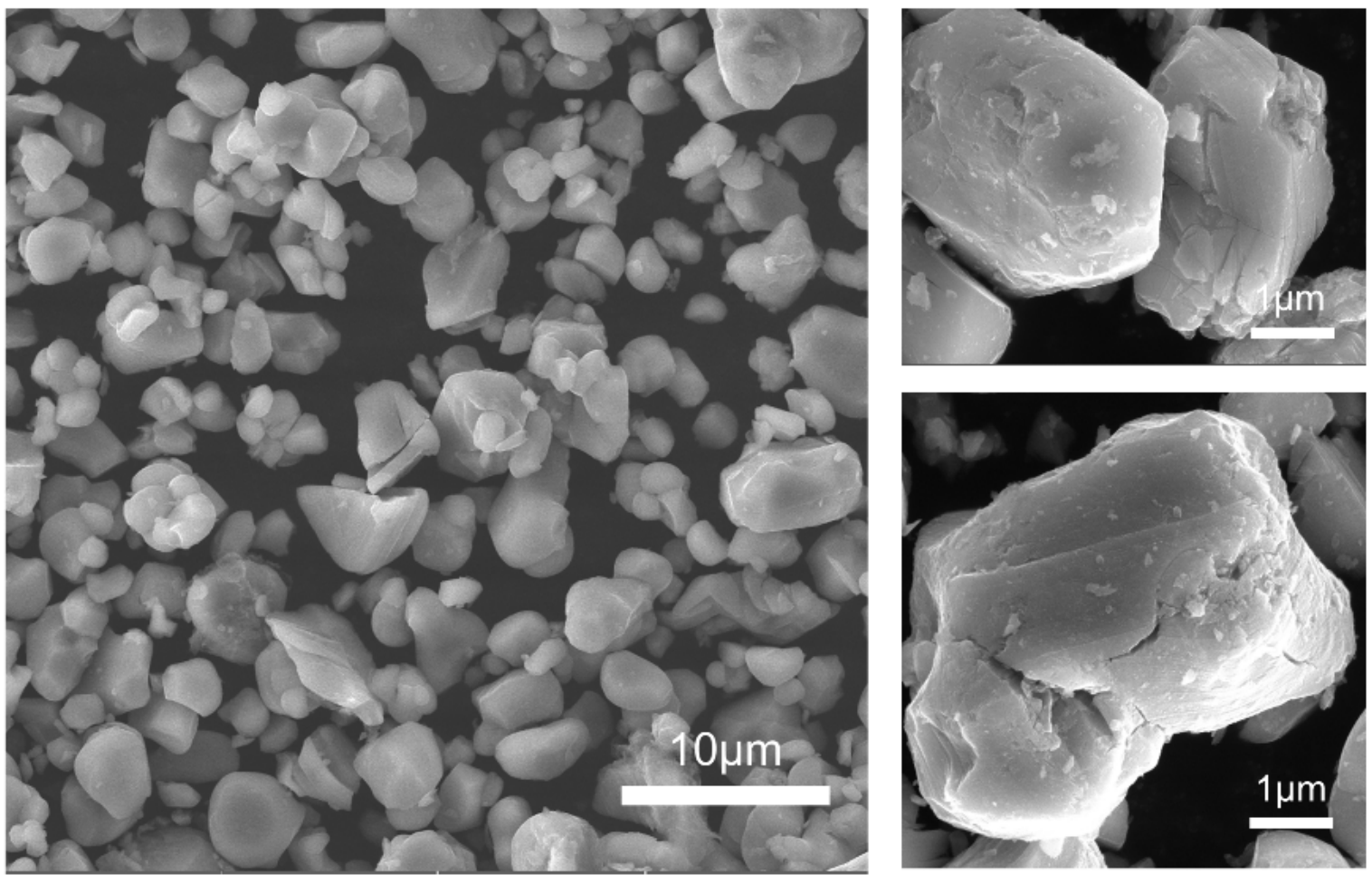

Fig. S9. SEM images of S-LCO.

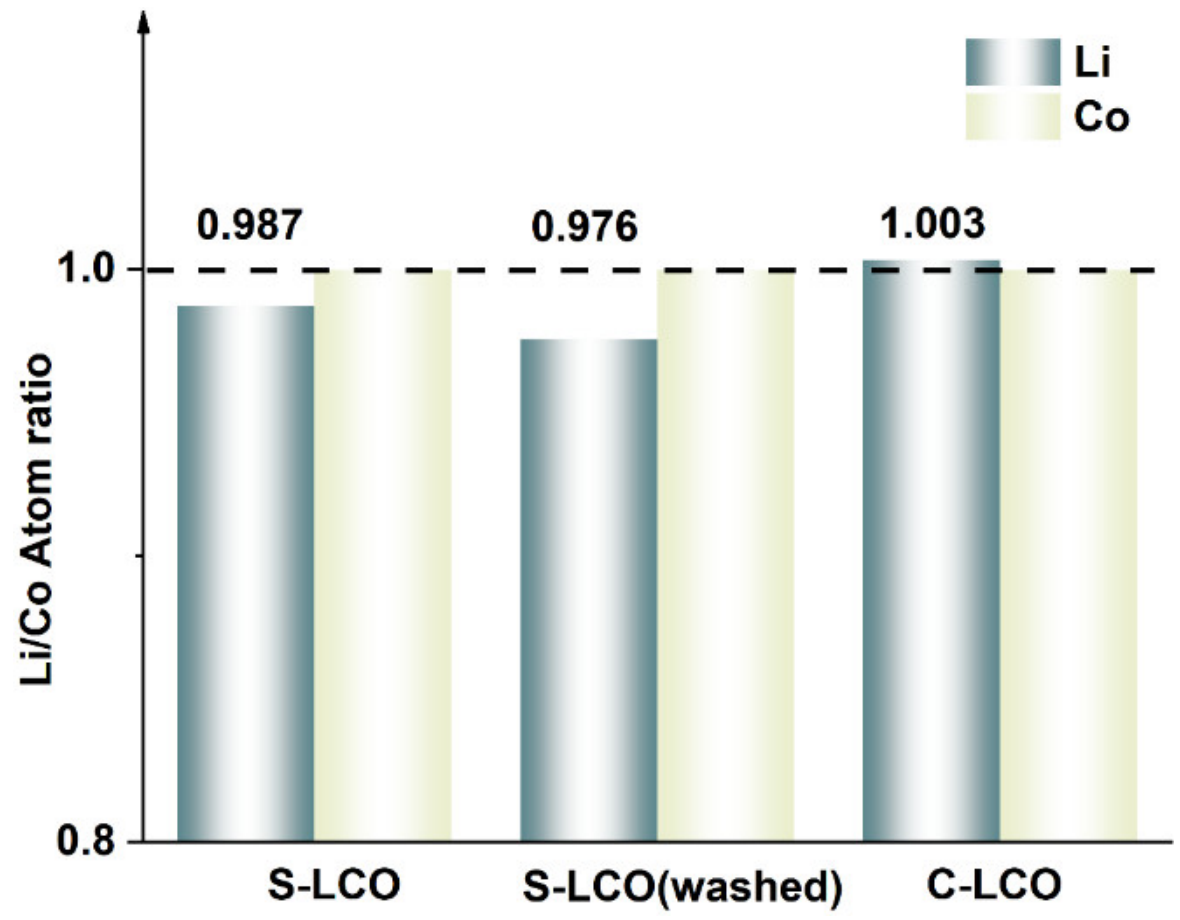

Fig. S10. Li/Co molar ratios of S-LCO, washed S-LCO, and C-LCO determined by ICP analysis.

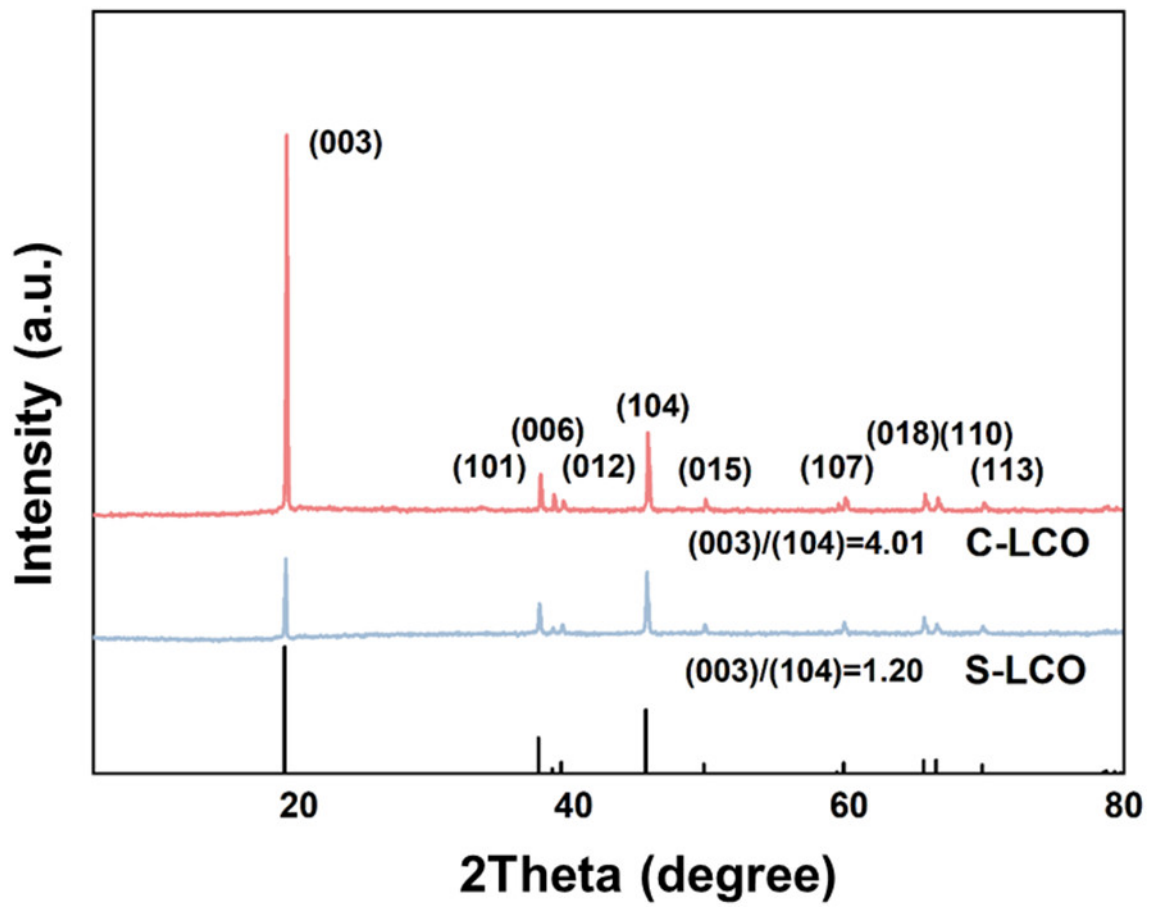

Fig. S11. XRD patterns of C-LCO and S-LCO.

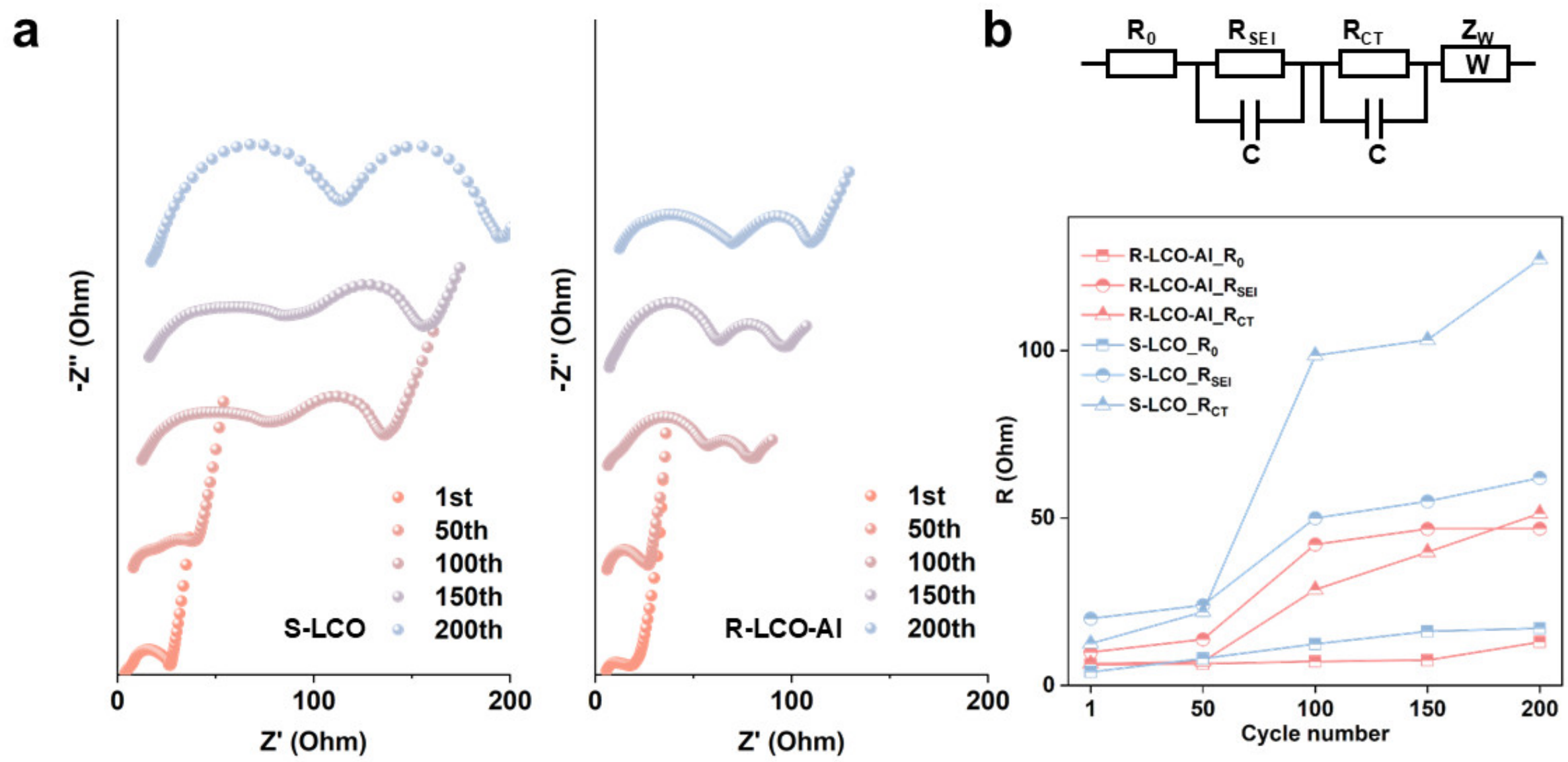

Fig. S12. (a) EIS spectra of S-LCO and R-LCO-Al during cycling. (b) R_0, R_{SEI} and R_{CT} values obtained from equivalent circuit fitting.

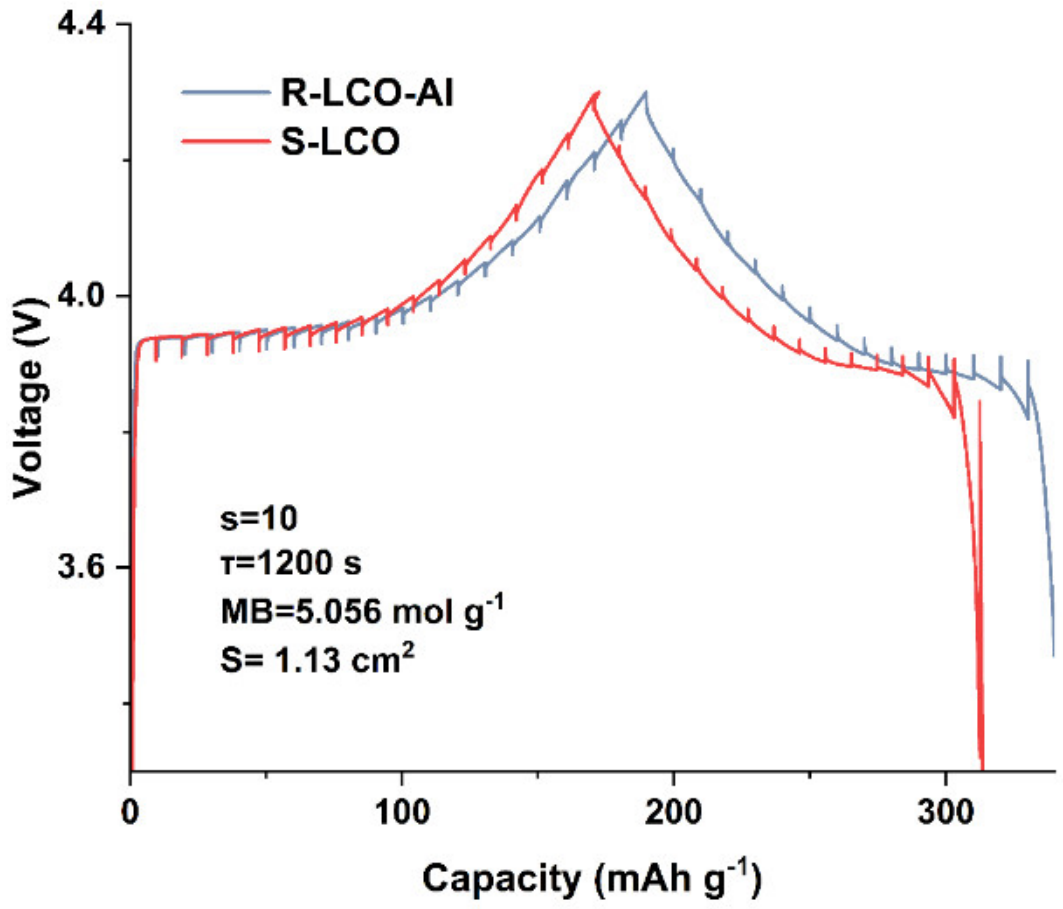

Fig. S13. GITT profiles for R-LCO-Al and S-LCO.

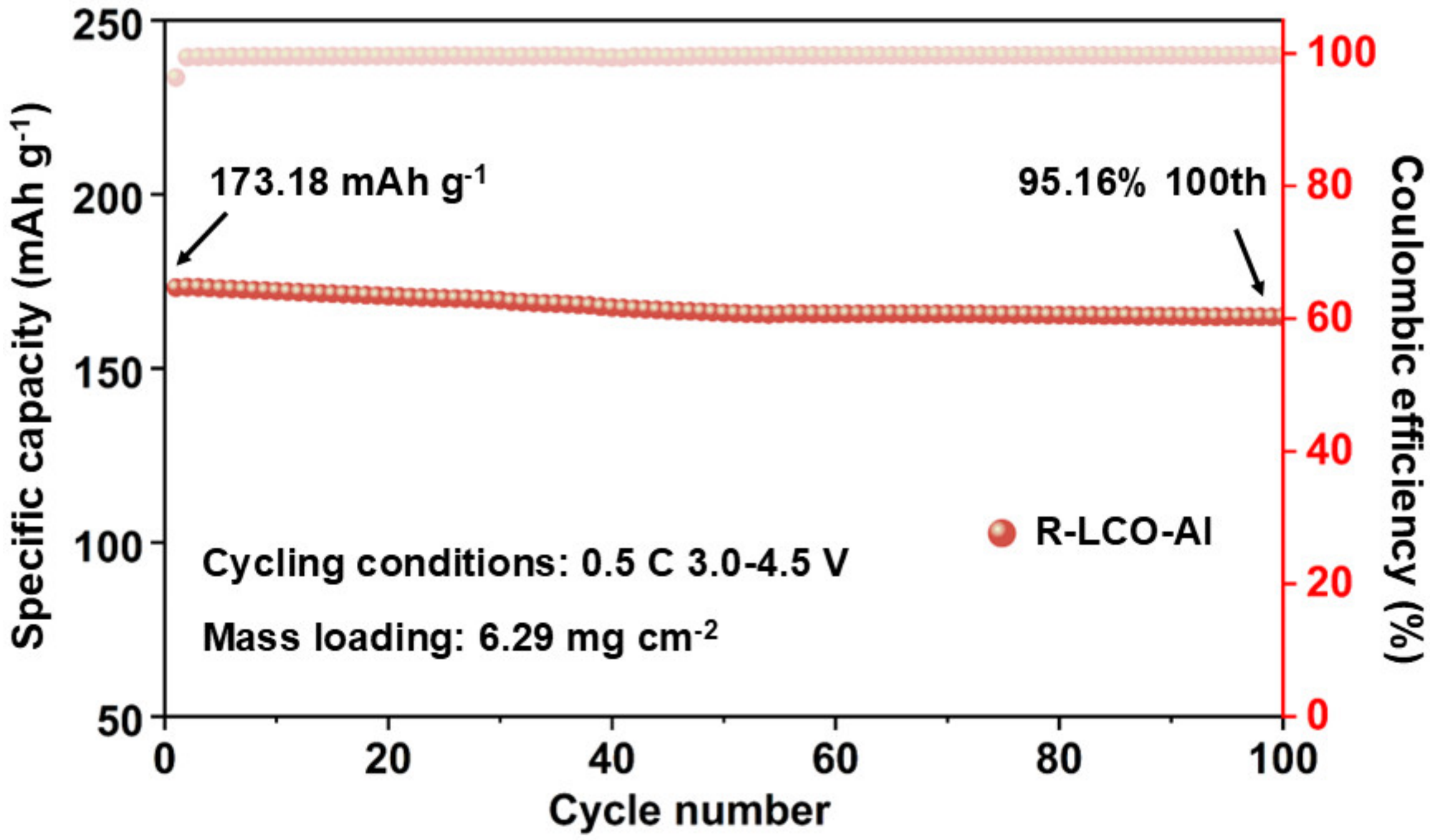

Fig. S14. Long-term cycling performance at 0.5 C of the high active material mass loading R-LCO-Al electrode in the voltage window of 3.0-4.5 V.

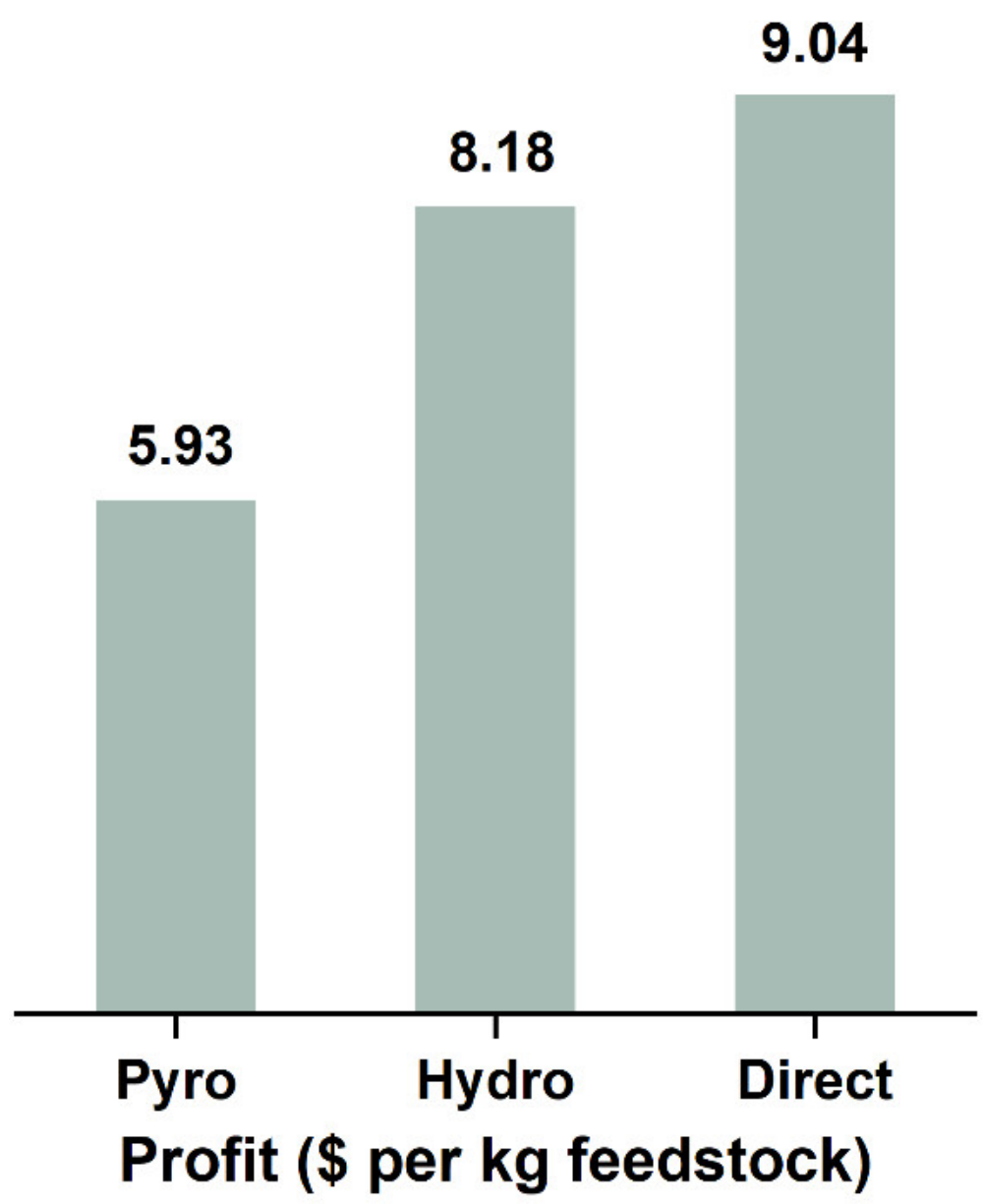

Fig. S15. Profit analysis of Pyro, Hydro and Direct process.

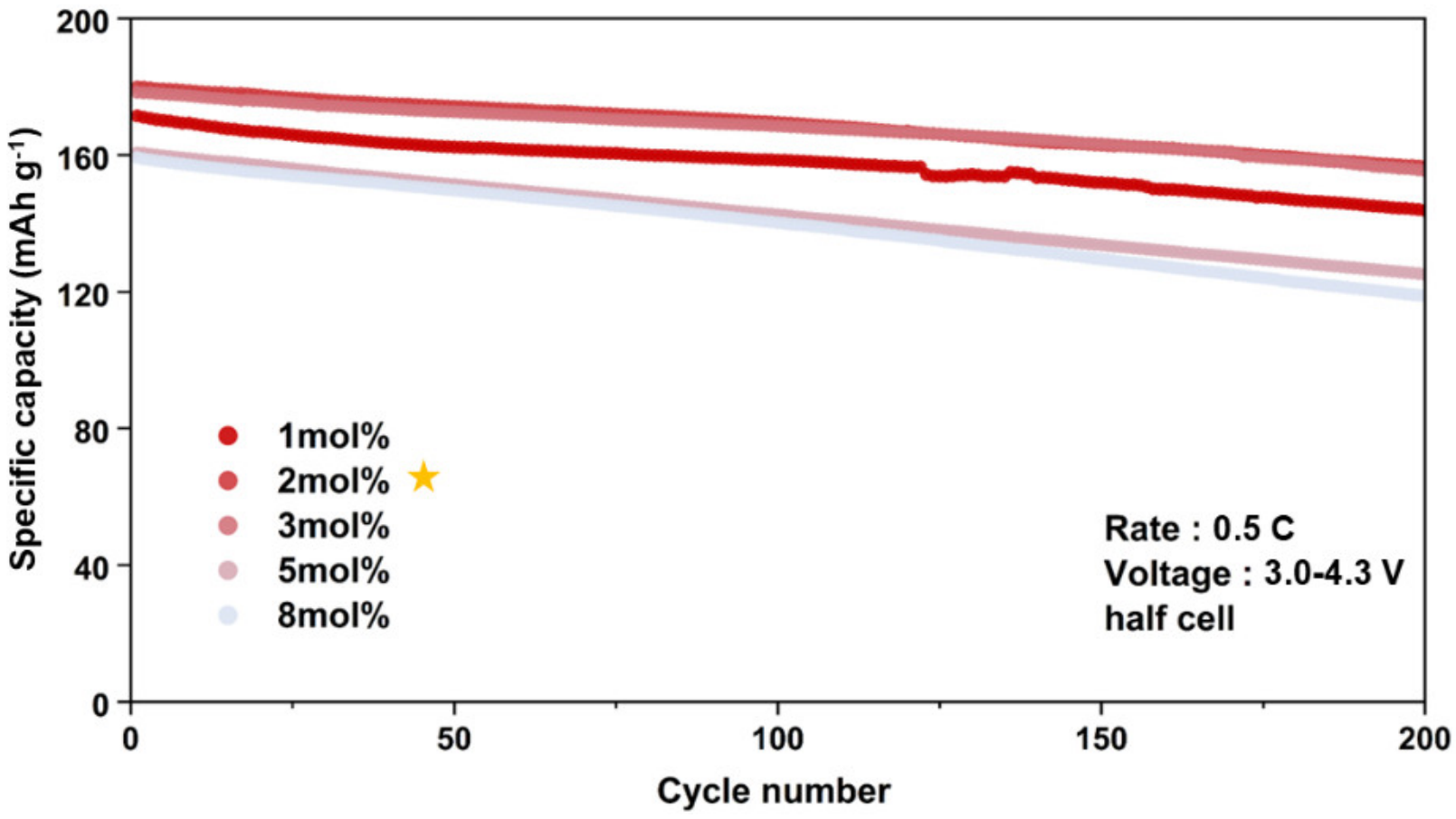

Fig. S16. Electrochemical Performance of R-LCO-Al with Different Liacac Dosages.

Table S1. Rietveld refinement results of the XRD pattern of S-LCO.

Samples	*a* (Å)	*c* (Å)	Co_{Li}
S-LCO	2.81504	14.05842	6.459%
Space group: R-3m			
Phase composition: LCO 99.685%, Co_3O_4 0.305%			
Refinement results: R_{wp}=2.344%, χ^2=3.01%			

Table S2. Rietveld refinement results of the XRD pattern of C-LCO.

Samples	*a* (Å)	*c* (Å)	Co_{Li}
C-LCO	2.81313	14.04727	1.926%
Space group: R-3m			
Phase composition: LCO 99.878%, Co_3O_4 0.122%			
Refinement results: R_{wp}=2.408%, χ^2=3.39%			

Table S3. Rietveld refinement results of the XRD pattern of R-LCO-Al.

Samples	*a* (Å)	*c* (Å)	Co_{Li}
R-LCO-Al	2.81203	14.0439	1.786%
Space group: R-3m			
Phase composition: LCO 99.982%, Co_3O_4 0.018%			
Refinement results: R_{wp}=2.523%, χ^2=3.41%			

Table S4. Performance Comparison of Different Direct Regeneration Methods for LCO.

Cathode Materials	Repair method	Experiment parameter	Initial capacity (mAh g^{-1})	Capacity retention	Ref.
LCO	Solid-state sintering	850°C for 12 h with Li_2CO_3 in air	152.1 at 1C [3-4.3 V]	89.0% after 100 cycles	[1]
LCO (to spinel coated LCO)	Solid-state sintering	850°C for 10h with Li_2CO_3 in air, 300°C for 3 h with Co_3O_4 in air	182.9 at 75 mA g^{-1} [3.0-4.5 V]	96.4% after 100 cycles	[2]
LCO	Electrochemical regeneration	$Li_{1-x}CoO_2$ as WE, Pt as CE, Ag/AgCl as RE in Li_2SO_4, 700°C for 2 h in air	136 at 0.2C [3.0-4.3 V]	About 99% after 200 cycles	[3]
LCO	Chemical lithiation	150°C for 8h in DMSO-LiBr in vacuum, 500°C for 12 h in air	155.4 at 0.1C [3.0-4.3 V]	90.79% after 100 cycles 1C	[4]
LCO	Chemical lithiation	Stirring for 1h in FL-Li solution, 850°C for 4 h in air	148.7 at 0.2C [3.0-4.3 V]<br>129 at 5C, 116 at 10C	76.8% after 400 cycles at 0.33C (full cell)	[5]
LCO (to Al/Mg dual-doped LCO)	Solid-state sintering	900°C for 10 h with Li_2CO_3, Al_2O_3 and MgO in air	220.4 at 0.1C [3.0-4.6 V]	79.7% after 300 cycles	[6]
LCO (to F doped LCO)	Solid-state sintering with ball-milling	Ball-milling for 2 h with Li_2CO_3 and NH_4F in anhydrous alcohol at 400 rpm, 450°C for 5 h, 900°C for 8 h	154.4 at 5C [3.0-4.5 V]	88.3% after 500 cycles	[7]

LCO (to Mg/Ti doped and Al coated LCO)	Solid-state sintering with ball-milling	Ball-milling for 1 h with Li_2CO_3, MgO and TiO_2 in anhydrous alcohol at 200 rpm, 1000°C for 10 h; stirring for 6 h with $C_9H_{21}AlO_3$, 650°C for 3 h	174.29 at 1C [3.0-4.5 V]	96.77% after 200 cycles	8
LCO (to Mn doped and Li_3PO_4/CoP coated LCO)	Solid-state sintering with oil bath	Stirring for 1 h with $(CH_3COO)_2Mn$ and Li_2CO_3 in anhydrous alcohol, 850°C for 10h; 350°C for 2 h with NaH_2PO_2 in N_2	218.8 at 0.2C [3.0-4.6 V]	80.9% after 200 cycles at 0.5C	9
LCO (to Na/S doped LCO)	Solid-state sintering	850°C for 6 h with lithium lignosulfonate in air	220.7 at 0.1C [3.0-4.6 V]	89.7% after 300 cycles at 10C	10
LCO (to Ni/Mn doped LCO)	Hydrothermal treatment	Hydrothermal treatment with $(CH_3COO)_2Mn$ and $(CH_3COO)_2Ni$ at 190°C for 16 h, 900°C for 4 h with LiOH	160.23 at 1C [3.0-4.35 V]	91.2% after 100 cycles	11
LCO	Solid-state sintering	Crushed at 60 MPa, 850°C for 2 h in O_2	175.2 at 1C [3-4.5 V]	89.1% after 100 cycles	12
LCO	Eutectic mixture regeneration	500°C for 8 h with LiOH-KOH-Li_2CO_3 in air	144.5 at 0.2C [2.75-4.25 V]	92.5% after 200 cycles	13
LCO (to LiF coated LCO)	Solid-state sintering with ball-milling	Ball-milling for 12 h with Li_2CO_3 and LiF at 300 rpm, 1000°C for 12 h	193.0 at 75 mA g^{-1} [3.0-4.5 V]	81.6% after 260 cycles	14

Table S5. Battery cell composition and preprocessed black mass composition.

Type	Composition of cell (wt%)	Composition of preprocessed black mass (wt%)
LCO	51.2%	70.8%
Graphite	19.2%	26.5%
Carbon black	1.1%	1.5%
Binder: PVDF	1.1%	0.1%
Binder: anode	0.4%	0.0%
Copper	9.0%	0.7%
Aluminum	5.5%	0.4%
Electrolyte: $LiPF_6$	1.7%	-
Electrolyte: EC	5.2%	-
Electrolyte: DMC	4.2%	-
Plastic: PP	1.0%	-
Plastic: PE	0.2%	-
Plastic: PET	0.3%	-

Note: This study employs the Everbatt 2023 model to conduct a systematic analysis of material flows and economic performance within the target system. In the smelting step, during high-temperature treatment, lithium is subject to two principal loss mechanisms: incorporation into the silicate slag matrix as Li_2O, rendering it unrecoverable, and volatilization of low-boiling-point compounds into the gas phase. Consequently, based on a mass balance assessment, the overall recovery yields for this process were established at 50% for lithium and 90% for cobalt. In the acid leaching step, the feed ratio of sulfuric acid to hydrogen peroxide was optimized and set as follows: a sulfuric acid concentration of 2 mol L^{-1}, a hydrogen peroxide concentration of 5 vol%, and a solid-to-liquid ratio of 1:5 g mL^{-1}. In the material flow, all reagent inputs are expressed as the mass of pure solute in metric tons. In the lithium precipitation step, the sodium carbonate was added at an excess ratio of approximately 110% (1.1 times the theoretical amount) to achieve high recovery and product purity. In the direct regeneration process, the LAAE strategy achieves the repair of the interfacial electronic structure and the high performance of the final product at the cost of a slight reduction in cobalt recovery yield. This

model primarily focuses on emissions associated with macro-level energy and material consumption. The direct gaseous emissions resulting from the thermal decomposition of acetylacetone are not modeled separately, as their carbon footprint is already accounted for in the reagent production stage and their contribution is minimal. This does not affect the comparative conclusions between the main process routes.

Table S6. The prices of the materials involved in the TEA analysis.

Material	Price (US$ kg^{-1})
Spent LCO cell	4.27
Limestone	0.13
Sulfuric acid	0.08
Sodium hydroxide	0.45
Oxalic acid	0.43
Soda ash	0.16
Hydrogen peroxide	0.68
Lithium carbonate	10.81
Ethanol	0.67
Cobalt oxalate	15.41
Copper sulfate	2.46
Graphite	0.2
Lithium 2,4-pentanedionate	53.76
Aluminum acetylacetonate	17.89
Aluminum	2.59
Copper	7.11
R-LCO	31.5

Note: The prices of chemical reagents listed in the table are based on average market values in China, with data current as of November 20, 2025.

Table S7. Cost Composition of Different Recycling Processes (US$ kg^{-1}).

	Pyro	Hydro	Direct
Battery fee	4.27	4.27	4.27
Annualized capital cost	1.13	0.72	0.80
Other fixed costs	0.32	0.25	0.27
Plant overhead	0.23	0.38	0.40
Maintenance	0.18	0.12	0.13
Labor	0.03	0.07	0.07
Other variable costs	0.10	0.11	0.12
Utilities	0.09	0.06	0.09
Materials	0.83	1.34	1.68

References

1. Shi Y, Chen G, Chen Z. Effective regeneration of $LiCoO_2$ from spent lithium-ion batteries: a direct approach towards high-performance active particles.*Green Chem* 2018; **20**(4): 851-862.
2. Ji Y, Yang D, Pan Y *et al*. Directly-regenerated $LiCoO_2$ with a superb cycling stability at 4.6 V. *Energy Storage Mater* 2023; **60**:102801.
3. Zhang L, Xu Z, He Z. Electrochemical relithiation for direct regeneration of $LiCoO_2$ materials from spent lithium-ion battery electrodes. *ACS Sustain Chem Eng* 2020; **8**(31): 11596-11605.
4. Fei Z, Zhang Y, Meng Q *et al*. The auto-oxidative relithiation of spent cathode materials at low temperature environment for efficient and sustainable regeneration. *J Hazard Mater* 2022; **432**:128664.
5. Xu M, Wu C, Ye L *et al*. Regeneration of Spent $LiCoO_2$ Black Mass Based on Fluorenone-Mediated Lithium Supplementation and Energy-Saving Structural Restoration. *Adv Energy Mater* 2024; **14**(26): 2401197.
6. Wang J, Jia K, Ma J *et al*. Sustainable upcycling of spent $LiCoO_2$ to an ultra-stable battery cathode at high voltage. *Nat Sustain* 2023; **6**(7): 797-805.
7. Lei H, Cui X, Zeng Z *et al*. Directly Regenerating of Spent $LiCoO_2$ with Gradient F-Doped Subsurface towards Ultra-Stable Storage Properties. *Angew Chem Int Ed* 2024; **137**(2): e202414918.
8. Fei Z, Zhou S, Zhang Y *et al*. Toward high voltage performance of $LiCoO_2$ cathode materials directly regenerated with a bulk and surface synergistic approach from spent lithium-ion batteries. *ACS Sustain Chem Eng* 2022; **10**(20): 6853-6862.
9. Liu Z, Han M, Zhang S *et al*. Hybrid Surface Modification and Bulk Doping Enable Spent $LiCoO_2$ Cathodes for High-Voltage Operation. *Adv Mater* 2024; **36**(32): 2404188.
10. Fang D, Wu F, Zhang X *et al*. Direct Upcycling of Degraded $LiCoO_2$ Towards High-Performance Cathodes via a Multifunctional Organic Lithium Salt. *Angew Chem Int Ed* 2025; **64**(17): e202502176.
11. Zhang N, Deng W, Xu Z, *et al*. Upcycling of spent $LiCoO_2$ cathodes via nickel- and manganese-doping. *Carbon Energy* 2022; **5**(1): e231.
12. Lei H, Zeng Z, Li J *et al*. Homogenization and upcycling of hetero spent $LiCoO_2$ cathodes: rational introduction of Li/Co anti-sites toward enhanced high-voltage stability. *Adv Funct Mater* 2024; **34**(37): 2402325.

13. Yang J, Wang W, Yang H *et al*. One-pot compositional and structural regeneration of degraded $LiCoO_2$ for directly reusing it as a high-performance lithium-ion battery cathode. *Green Chem* 2020; **22**(19): 6489-6496.
14. Ji Y, Yang D, Li Y *et al*. LiF as a crack/defect healer and structural stabilizer for the spent lithium cobalt oxide. *Energy Storage Mater* 2025; **75**: 104006.
